# Enhanced Lattice Coherences and Improved Structural Stability in Quadruple A‐Site Substituted Lead Bromide Perovskites

**DOI:** 10.1002/smll.202500977

**Published:** 2025-04-18

**Authors:** Marie Cherasse, Niusha Heshmati, Joanna M. Urban, Feray Ünlü, Michael S. Spencer, Maximilian Frenzel, Luca Perfetti, Sanjay Mathur, Sebastian F. Maehrlein

**Affiliations:** ^1^ Fritz Haber Institute of the Max Planck Society Department of Physical Chemistry Berlin Germany; ^2^ LSI CEA/DRF/IRAMIS CNRS Ecole Polytechnique Institut Polytechnique de Paris Palaiseau France; ^3^ Institute of Inorganic and Materials Chemistry Department of Chemistry University of Cologne 50939 Cologne Germany; ^4^ Department of Solution‐Processed Materials and Devices Hysprint Innovation Lab Helmholtz‐Zentrum Berlin für Materialen und Energie GmbH Berlin Germany; ^5^ Helmholtz‐Zentrum Dresden‐Rossendorf Institute of Radiation Physics Dresden Germany; ^6^ Technische Universität Dresden Institute of Applied Physics Dresden Germany; ^7^ Present address: Laboratoire d'Optique Appliquée ENSTA Paris CNRS Ecole Polytechnique Institut Polytechnique de Paris Palaiseau 91761 France

**Keywords:** A‐site cation engineering, coherent phonons, lattice dynamics, lead halide perovskites, nonlinear THz spectroscopy, optoelectronic properties, structural phase stability

## Abstract

Lead halide perovskites (LHPs) are promising materials for efficient photovoltaic devices; however, they often encounter limited structural stability and degradation problems that limit their technological potential. This study investigates a novel perovskite composition consisting of (Cs, MA, FA, GA)PbBr_3,_ abbreviated as (4cat)PbBr_3_, to effectively enhance phase stability and optoelectronic characteristics. The spectroscopic data reveal improved structural order, electronic properties, and dynamic lattice response in a cubic phase, which is uniquely stabilized by the specific cation composition down to 80 K. Superior optoelectronic properties are verified by increased photoluminescence (PL) and 20‐fold higher electron mobility, when compared to the single‐cation composition, MAPbBr_3_. Notably, the ultrafast Terahertz‐induced Kerr effect (TKE) reveals a dominating 1.1 THz octahedral twist mode, also observed in MAPbBr_3_, however with a doubled phonon coherence time in (4cat)PbBr_3_ at 80 K. The observation of higher structural order in the 4‐cation composition is thus reflected by the prolonged lattice coherences, indicating enhanced dynamic screening effects that can explain the improved optoelectronic properties of (4cat)PbBr_3_. This study therefore sheds light on the influence of the A‐site cation composition on the inorganic sublattice and its coherent dynamics, highly relevant to perovskite‐based photovoltaic and optoelectronic technologies.

## Graphical abstract

This study explores a novel perovskite composition, (4cat)PbBr_3_, which combines four A‐site cations to improve stability and optoelectronic properties. Compared to MAPbBr_3_, this new composition shows better structural order, higher photoluminescence, and better electron mobility. Key findings include a unique stabilization of its cubic phase at low temperatures and extended phonon coherence times, suggesting enhanced performance for optoelectronic applications.

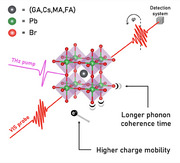

## 1 Introduction

Hybrid perovskites combine the advantages of cost‐efficient production and versatile processing with superior charge‐transport properties and efficient band‐gap tuning. This leads to excellent optoelectronic properties for photovoltaic and electroluminescent applications, ^[^ ^1^ ^]^ requiring high charge mobility, long carrier diffusion lengths, and exceptional photoluminescence efficiency.^[^ ^2^ ^]^ However, the degradation of the perovskite material and gradual transition to a non‐photoactive *δ*‐phase due to ambient exposure are current obstacles on their path to industrial applications.^[^ ^3^ ^]^ Nevertheless, the high chemical tunability of perovskite compositions in conjunction with surface engineering, ^[^ ^4^ ^]^ offers unique perspectives to counteract the intrinsic challenges of limited structural and environmental stability.^[^ ^5^ ^]^ Recent studies reveal that the optoelectronic properties of multi‐component lead halide perovskites are influenced by their crystal lattice symmetry and orbital overlap.^[^ ^6^ ^]^ The cubic phase typically demonstrates superior charge mobility and longer diffusion lengths, enhancing charge transport in perovskite solar cells.^[^ ^7^, ^8^ ^]^ Nevertheless, the higher formation enthalpy contribution to the overall Gibbs energy, makes the cubic (*α*) phase vulnerable to transformation into a non‐photoactive hexagonal (*δ*) phase at low temperatures.^[^ ^9^ ^]^

Altered compositions within the ABX_3_ crystal system have been explored in previous studies to address these structural challenges.^[^ ^5^, ^10^ ^]^ The size of cations and hydrogen bonding mechanisms play a crucial role in lattice stability^[^ ^11^ ^]^ whereas altering the A‐site cation enhances interactions within the BX_6_ octahedra, changing local and dynamic crystal lattice disorder at room temperature.^[^ ^12^, ^13^ ^]^ In this context, it has been observed that smaller ionic radii of halide anions (X^−^) contribute to the stabilization of the cubic structure thereby enhancing photostability by suppressing A‐site cation diffusion.^[^ ^14^, ^15^ ^]^

The underlying mechanisms of the outstanding optoelectronic properties in hybrid perovskites are often attributed to a high defect tolerance, potentially involving dynamic charge carrier screening.^[^ ^16^, ^17^ ^]^ The latter hinges on the electron‐phonon coupling, dictated by the structure and dynamics of the lead‐halide framework.^[^ ^18^, ^19^, ^20^ ^]^ At room temperature, A‐site cations in hybrid ABX_3_ (A: Methylammonium(MA), Formamidinium(FA), Guanidinium(GA), or Cs) halide perovskites contribute to dynamic structural disorder.^[^ ^21^, ^22^ ^]^ In conjunction with the highly polar and anharmonic lattice, this may play a pivotal role in influencing their optoelectronic properties by dynamic screening of charge carriers.^[^ ^23^, ^24^, ^25^ ^]^ Therefore, the mutual influence of A‐site cation and inorganic sublattice, as well as the interplay between lattice dynamics and charge carriers has to be carefully considered when optimizing LHP compositions for durability under operating conditions. To this end, the precise mechanisms governing the interaction between charge carriers and the highly polarizable and anharmonic LHP lattice remain subjects of ongoing investigations.^[^ ^20^, ^26^, ^27^ ^]^ Cutting‐edge experimental techniques, such as ultrafast time‐resolved electron emission^[^ ^28^ ^]^ and nonlinear terahertz spectroscopy, ^[^ ^20^, ^29^ ^]^ have been instrumental in shedding light on the rapid charge screening and the ultrafast polarizability of the lead halide lattice. These methods thus allow probing of the carrier and lattice dynamics on picosecond time scales, providing valuable insights into the temporal evolution of charge carriers in hybrid perovskites.^[^ ^20^, ^28^ ^]^

This work explores A‐site cation engineering to improve the structural and optoelectronic properties of A‐site substituted quadruple‐cation perovskite ((Cs, MA, FA, GA)PbBr_3,_ abbreviated as (4cat)PbBr_3_) single crystals and thin films. To elucidate the intermixing of dynamic and static, lattice and charge carrier properties, we apply a state‐of‐the‐art combination of techniques. The equilibrium properties of as‐synthesized LHPs with respect to the chemical composition and Goldschmidt tolerance factor *t* provide information about the crystal structure stability and defects. In addition, thin film surface morphology and optoelectronic properties including absorbance, photoluminescence, and electron mobility are explored in (4cat)PbBr_3_ and compared to the parent compound MAPbBr_3_.

The dynamic lattice properties of LHPs are investigated by the Terahertz‐induced Kerr effect, which has been recently employed to obtain coherent control over the hybrid perovskite lattice via nonlinear excitation pathways.^[^ ^20^ ^]^ In MAPbBr_3_, this technique allowed for coherent control over the 1.1 THz octahedral twisting mode, ^[^ ^20^ ^]^ which strongly modulates the electronic bandgap, ^[^ ^30^ ^]^ simultaneously acts as the order parameter for a structural phase transition, ^[^ ^31^, ^32^ ^]^ and significantly contributes to dynamic disorder.^[^ ^22^, ^33^ ^]^ In the low temperature orthorhombic phase, this mode was found to dominate the phonon‐modulated THz polarizability with potential implications for dynamic charge carrier screening beyond the Fröhlich polaron model.^[^ ^16^, ^20^ ^]^ Hence, we here assess the influence of A‐site cation composition on the dynamic lattice polarizability as a response to a single‐cycle THz electric field spike. To accomplish this, several conventional and complex A‐site cation LHPs were studied by the ultrafast TKE. By this, we reveal a counter‐intuitive higher dynamic order in quadruple‐cation mixed compounds compared to single A‐site LHPs. This manifests as prolonged lattice coherence in the (4cat)PbBr_3_ perovskite, potentially contributing to the higher radiative recombination and several fold (20‐times) improved electron mobility, when compared to the reference composition MAPbBr_3_.

## 2 Results

### 2.1 Design, Synthesis, and Morphology

**Figure 1:**
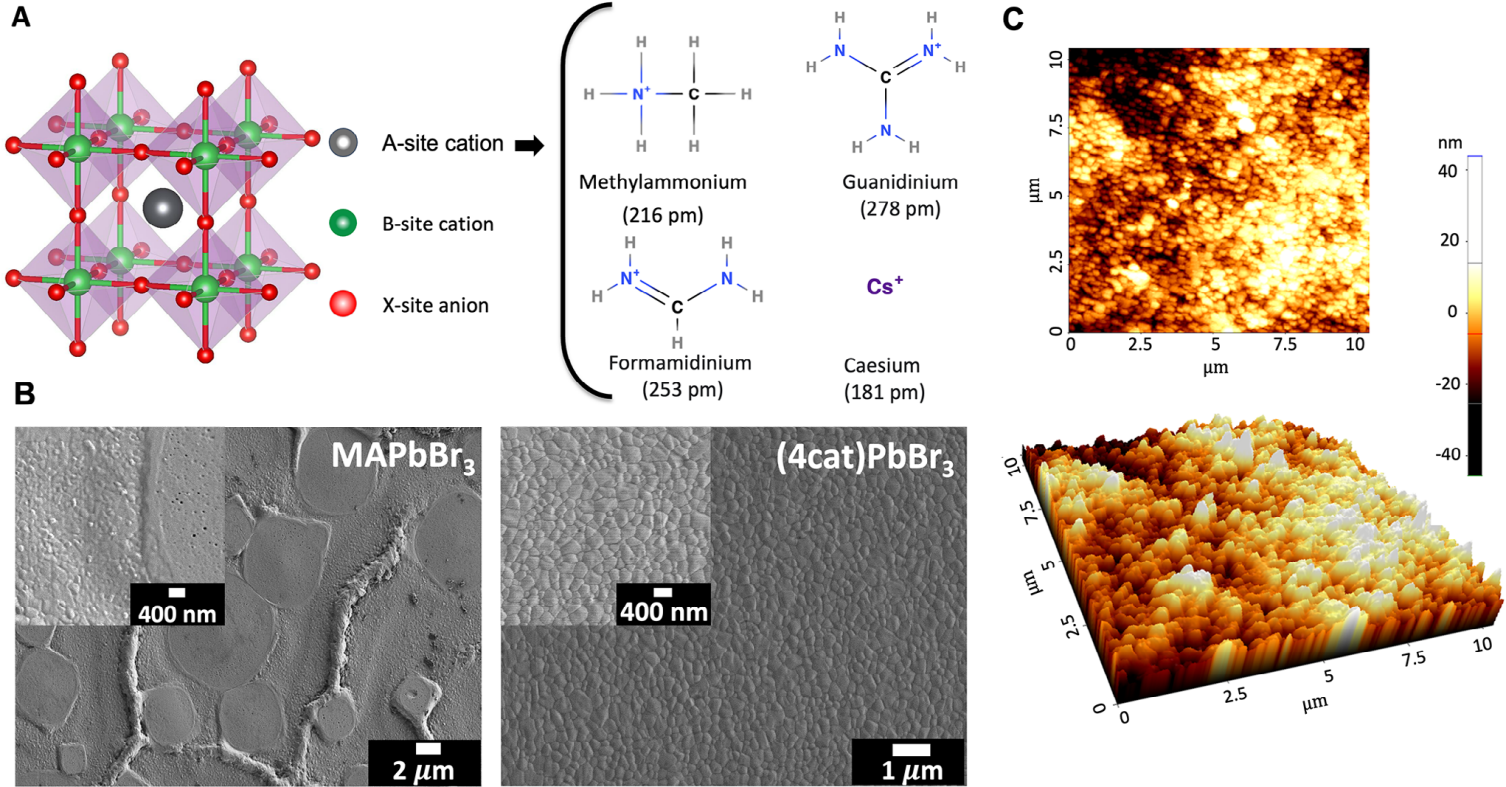
Structure and surface properties A) ABX_3_ perovskite crystal structure and chemical structure of incorporated A‐site cations used in the synthesis of the (4cat)PbBr_3_. B) SEM images of MAPbBr_3_ and (4cat)PbBr_3_ perovskite films. (4cat)PbBr_3_ shows full coverage, uniform grain size distribution, and a pinhole‐free surface evaluated at room temperature. C) The non‐contact (NC) height mode AFM image reveals a root mean square (RMS) roughness of 10 nm, indicating a smooth surface at room temperature.

First, we focus on the design, synthesis, and characterization of novel LHP thin films with enhanced stability, optoelectronic properties, and surface morphology (Section S1, Supporting Information). The investigation involves compositional engineering of A‐site cations, guided by optimizing the geometrical tolerance factor (*t*) with optimal *t* = 1 to achieve a robust cubic crystal structure (Section S2, Supporting Information).^[^ ^34^ ^]^ The ABX_3_ lattice structure in 3D LHPs restricts the acceptable size range for the A‐site cation.^[^ ^34^ ^]^ Exceeding this range leads to lower‐dimensional structures, while smaller cations cause lattice strain, hindering a stable 3D structure.^[^ ^35^ ^]^ This phenomenon can be captured by the tolerance factor as detailed in the Table S1, Supporting Information. **Figure** **1**A shows the perovskite lattice structure alongside the four distinct A‐site cations employed. We specifically design the chemical composition (GA_0.015_Cs_0.046_ MA_0.152_FA_0.787_)PbBr_3_, which optimizes the tolerance factor to *t* = 0.99, to achieve the most stable cubic crystal structure.

Thin films of MAPbBr_3_ and (4cat)PbBr_3_ with an average thickness of 350 nm were fabricated and analyzed by scanning electron microscopy (SEM) and topographic atomic force microscopy (AFM). The in plane SEM analysis of a MAPbBr_3_ film in Figure 1B (left panel) shows a rough topography constituted by nano‐ and micro‐grains, which is known to be detrimental for the device performance (see Section S3, Supporting Information).^[^ ^36^ ^]^ In contrast, the surface morphology of the (4cat)PbBr_3_ films (Figure 1B, right panel), exhibited a uniform morphology formed by dense microstructure and nearly defect‐free surface with a grain size of ≈200 nm and a low surface roughness of 10 nm (see Figure 1C).

### 2.2 Optoelectronic Characterization

**Figure 2:**
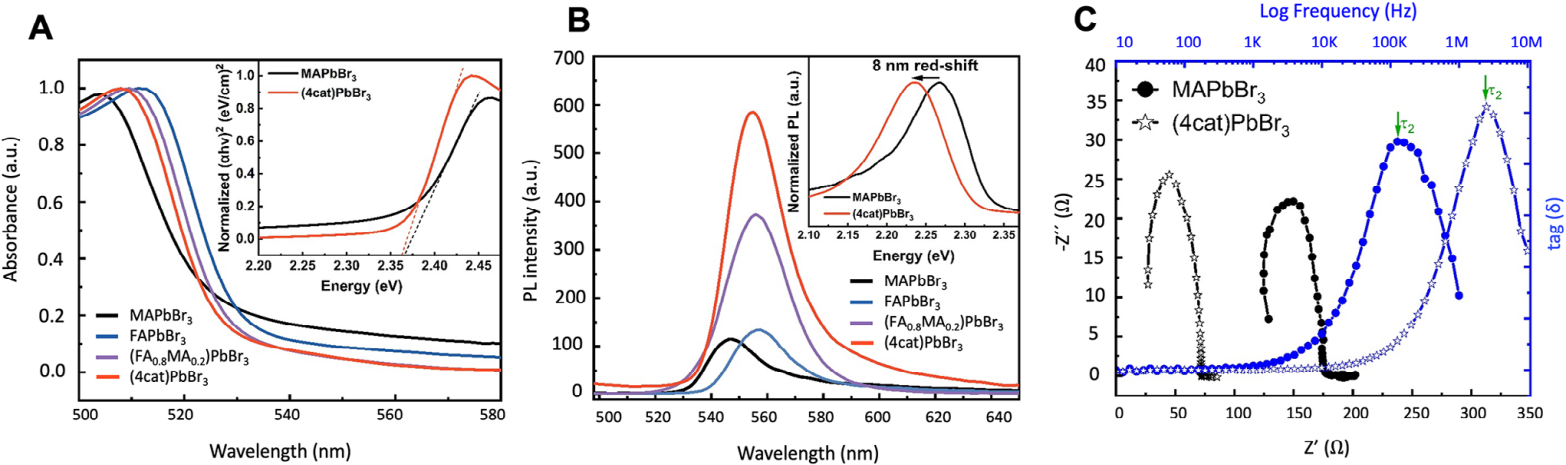
Optical and charge transport properties comparing various LHP thin films: A) Absorption spectra of LHP thin films and Tauc plots (inset). The direct optical band gaps of perovskite thin films are determined from the Tauc plots fit to ≈ 2.36 eV and 2.37 eV for the (4cat)PbBr_3_ and benchmark MAPbBr_3_ LHP. B) PL at 300 K with excitation wavelength at 450–460 nm for various thin films shows highest emission for the (4cat)PbBr_3_ thin film, indicating the radiative efficiency increases with multiple A‐site cations. C) Impedance spectroscopy measurements using a simple RC model (FTO/Perovskite/gold): Comparison of MAPbBr_3_ (dot markers) and (4cat)PbBr_3_ (star markers) with 350 nm thickness. (4cat)PbBr_3_ shows more than twenty times higher diffusion coefficient and electron mobility compared to MAPbBr_3_.

The impact of A‐site cation engineering on the light absorption and radiative recombination behavior was studied by ultraviolet–visible spectroscopy (UV‐VIS) and photoluminescence (PL) emission spectroscopy that were compared to various benchmark thin film samples at room temperature. All thin films exhibited a distinct absorption onset at 530–535 nm (see **Figure** **2**A), which varies by few nm in wavelength for different compositions.^[^ ^37^ ^]^ The spectra for FAPbBr_3_, (FA_0.8_MA_0.2_) PbBr_3_, and (4cat) PbBr_3_ perovskite exhibit almost similar absorbance suggesting a correlation with the surface properties of the films (Figure S3, Supporting Information).

The optical bandgap (*E*_g_) was estimated from the spectral data as around *E*_g_ = 2.36 for (4cat)PbBr_3_ eV, and *E*_g_ = 2.37 eV for MAPbBr_3_, using a usual relationship between the absorption coefficient and the energy of incident photons.^[^ ^38^ ^]^ Figure 2B unveils significantly enhanced room temperature steady‐state PL for (4cat)PbBr_3_, three times higher than the MAPbBr_3_ parent compound under the same measurement condition. Compared to MAPbBr_3_, the (4cat)PbBr_3_ emission peak is red‐shifted from 2.27 (547 nm) to 2.23 eV (555 nm). In comparison to (4cat)PbBr_3_ with FWHM ≈ 24.3 nm, the broader emission peak for MAPbBr_3_ (FWHM ≈ 25.1 nm) can be attributed to distorted octahedra and dynamic disorder affecting the electronic structure.^[^ ^39^ ^]^ Moreover, the Urbach energy extracted from the absorbance data shows a similar relative trend, increasing from 58 ± 5 meV for (4cat)PbBr_3_ film to 83 ± 5 meV for MAPbBr_3_ (See Section S4, Supporting Information). Therefore, higher disorder increases both the Urbach energy and the broadening of the PL peak.^[^ ^40^ ^]^ Additionally, the narrower peak also implies a more uniform crystal structure, meaning that the composition and structure are more homogenous throughout the material.^[^ ^41^ ^]^ Complementary impedance spectroscopy (IS) provides insights into the charge transport properties and the respective diffusion coefficient.^[^ ^42^ ^]^ Here, we prepared devices with an FTO/Perovskite/Au structure (see Figure S5, Supporting Information). The characteristic Nyquist plots for MAPbBr_3_ and (4cat)PbBr_3_ are shown in Figure 2C. By simple modelling the electrical equivalent circuit (EEC) ^[^ ^43^, ^44^ ^]^ (see Section S5 and Table S2, Supporting Information), we estimate twenty‐fold increase in electron mobility for (4cat)PbBr_3_ (0.75 cm^2^ V^−1^ s^−1^) compared to MAPbBr_3_ (0.037 cm^2^ V^−1^ s^−1^), suggesting fewer traps along charge transport pathways in the perovskite bulk. A similarly higher diffusion coefficient was also observed for mixed halide quadruple cation perovskites by Jung et al., where the value of 0.05 cm^2^ s⁻¹ was obtained by a transient grating method.^[^ ^45^ ^]^ This is consistent with the narrower PL spectral width and steeper absorption coefficient for (4cat)PbBr_3_ perovskite. As discussed in Section S5 (Supporting Information), the devices were prepared with an FTO/Perovskite/Au architecture and no charge selective material was used. Therefore, the calculated mobilities are lower than when charge selective materials facilitate the process.

### 2.3 Ultrafast Lattice Dynamics

**Figure 3:**
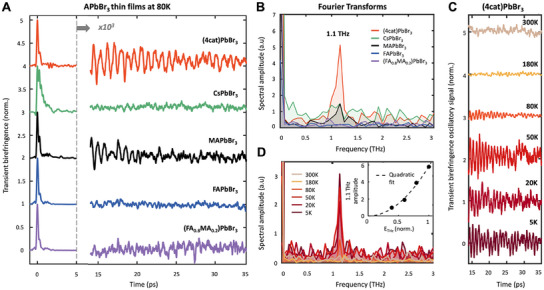
Coherent phonon dynamics of LHP thin films via Terahertz‐induced Kerr effect (TKE): A) Comparison of THz‐induced birefringence in various A‐site cation lead bromide perovskites; all ≈350 nm thin films on 500 µm BK7 glass substrate at 80K. Left hand section: First 5 ps responses normalized to the instantaneous electronic responses (at *t* = 0). Right hand section: Long lived signals (>14 ps), rescaled by factor 10^3^. B) FTs of the long‐time TKE responses after 14 ps. C) Long‐lived oscillatory TKE features of the (4cat)PbBr_3_ thin film as a function of temperature (normalized to *t* = 0 peak signal and offset by 1 for each curve). D) FTs of the temperature dependent (4cat)PbBr_3_ TKE responses in C. Inset: THz field dependence of the 1.1 THz peak at 80 K, including fit to quadratic dependence on the THz electric field peak *E*_THz_.

The structural properties of (4cat)PbBr_3_ are particularly relevant to understand the role of lattice dynamics for dynamic carrier screening, which impacts the charge carriers’ mobility and their lifetimes.^[^ ^16^, ^17^ ^]^ We therefore investigated the lattice dynamics and its coherence in the presence of complex mixing of multiple cation species. **Figure** **3**A, shows the impact of the A‐site cation's chemical composition on the THz‐induced Kerr effect (TKE) for 350 nm‐thick, thin films at 80 K. During the first ≈5 ps (left panel), the normalized THz‐induced birefringence is dominated by the instantaneous electronic polarizability, giving rise to peak signal at *t* = 0 (Section S6, Supporting Information).^[^ ^20^ ^]^ The rescaled long‐lived response (right panel, normalized to the respective *t* = 0 peak signal), reveals only oscillatory features in MAPbBr_3_ and (4cat)PbBr_3_ compounds. Recently, these features were attributed to coherent phonons excited via a strong nonlinear THz polarizability that provided a direct measure of lattice coherence times in MAPbBr_3_.^[^ ^20^ ^]^ The modes were identified as octahedral twist modes in the MAPbBr_3_ parent compound and were found to be highly dependent on the A‐site cation species, which potentially forms additional H‐bonds.^[^ ^46^, ^47^ ^]^ Substituting 80% or more of MA^+^ with FA^+^, leads to a full suppression of any observable long‐lived lattice coherence (see Figure 3A).

The suppression of long‐lived coherence becomes even more obvious in Figure 3B, which displays the Fourier transform (FT) of the long‐time scale TKE signals (right section of Figure 3A). For MAPbBr_3_ and (4cat)PbBr_3_, the long‐lived oscillatory signals are dominated by a single mode at 1.1 THz, which strongly depends on the A‐site cation's chemical composition, both in amplitude and relative spectral width. Considering the minority MA^+^ (15%) and majority FA^+^ (79%) content, it is even more remarkable that the cubic (4cat)PbBr_3_ mimics the lattice response of orthorhombic MAPbBr_3_ and not the one of (FA_0.8_MA_0.2_)PbBr_3_, which highlights the intriguing role of the GA^+^ cations. In contrast to an expected lattice distortion and increased dynamic disorder from the variety of cation sizes, the phonon lifetime appears to be longer in (4cat)PbBr_3_ (see Figure 3B). Our detailed analysis (Section S7, Supporting Information) shows almost a doubled phonon lifetime (*τ_4cat,80K_* = 6.5 ps ± 0.9) of the octahedral twist mode compared to MAPbBr_3_ at 80K (*τ_MA,80K_* = 3.7 ps ± 0.7). This suggests that the compositionally complex (4cat)PbBr_3_ perovskite offers a surprisingly higher structural ordering and less lattice anharmonicity, beneficial for concerted lattice dynamics. Therefore, adding GA^+^ and Cs^+^ cations in the ideal Goldschmidt ratio mentioned above, strikingly not only restores, but even doubles the lattice coherence time.

The temperature dependence of the long‐lived lattice coherence was investigated by TKE experiments conducted from 300 K down to 5 K (Figure 3C). Cooling to such low temperatures further reduces phonon damping and facilitates the observation of sharper phonon features, e.g. for the investigation of potential mode splitting or short‐lived superimposed responses (< 14 ps) (see Experimental Section/Methods). Notably, clear 1.1 THz oscillatory features appear at 80 K (Figure 3B), and become more prominent as the temperature decreases to 5 K. Their FTs in Figure 3D unveil a continuous narrowing of the 1.1 THz peak as the temperature approaches 5 K, providing further proof of the oscillatory signal originating from a coherent lattice motion. Nevertheless, down to 5 K, no mode splitting is observed, which strongly supports the absence of any structural phase transition. The inset of Figure 3D displays the excitation field‐dependence of the 1.1 THz oscillation in (4cat)PbBr_3_ at 80 K. It unveils a scaling with the square of the THz electric field amplitude, suggesting a nonlinear excitation mechanism with a Raman‐type driving force,^[^ ^20^, ^48^, ^49^ ^]^ and providing clear evidence that we indeed observe nonlinear excitation of 1.1 THz Raman‐active phonon modes in (4cat)PbBr_3_ (Section S8, Supporting Information), as observed in MAPbBr_3_.^[^ ^20^ ^]^ Notably, the samples showed excellent reproducibility and stability of the oscillatory response across various sample locations, which rules out phase segregations or other inhomogeneities in the samples. Measurements were performed at intervals of four months, and the results confirm the robust stability of the (4cat)PbBr_3_ thin film (Section S9, Supporting Information).

### 2.4 Temperature‐Dependent Phase Stability and Phonon Lifetimes

**Figure 4:**
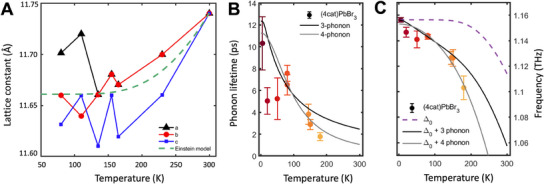
Temperature dependent static and dynamic lattice properties A) Temperature‐dependent XRD of (4cat)PbBr_3_ single crystal confirms the presence of cubic phase perovskite down to 80K. Dashed line: Einstein model for average lattice constant. B) Phonon lifetime and C) Phonon frequency of the 1.1 THz mode of (4cat)PbBr_3_ in the low temperature range. Details: B) fit to anharmonic decay model assuming three‐phonon (black line) and four‐phonon scattering (grey line) C) Frequency shift of the 1.1 THz mode Δ_0_ due to lattice expansion based on thermal expansion coefficient obtained from the Einstein model fit in A (dashed purple line). Solid lines: total shift due to contributions from lattice expansion and three‐phonon scattering (black) and four‐phonon scattering (grey).

To confirm the structural phase stability indicated by the absence of mode splitting in Figure 3D, structural phase transitions above 80 K are additionally ruled out by temperature‐dependent single crystals X‐ray diffraction (XRD) up to 300 K. Previously, it has been reported that the stability of the cubic phase for FAPbBr_3_ extends down to 170 K.^[^ ^50^, ^51^ ^]^ **Figure** **4**A exhibits the unique structural properties of (4cat)PbBr_3_ single crystals, maintaining a crystalline cubic phase down to 80 K, in conjunction with a slight contraction with decreasing temperature.^[^ ^45^ ^]^ This further confirms our hypothesis, that a specifically tailored composition of (4cat)PbBr_3_ can effectively suppress phase transitions observed in the parent MAPbBr_3_ compounds. This finding is additionally supported by Raman spectroscopy on (4cat)PbBr_3_ single crystals across temperatures from 300 to 80 K: The results in Figure S11C (Supporting Information) only show slight temperature‐dependent peak narrowing while maintaining Lorentzian shapes down to 80 K (Section S10, Supporting Information).

Finally, the observed temperature dependence of the average cubic lattice constant was modeled using the Einstein model (Section S11, Supporting Information).^[^ ^52^ ^]^ Based on the fit in Figure 4A (dashed line), we estimate the thermal expansion coefficient that is used to calculate the phonon mode shift due to lattice expansion (Section S12, Supporting Information). We directly determine the lifetime of the 1.1 THz mode as a function of temperature by time‐domain fits (Section S7, Supporting Information). For this, the observed decay of the phonon amplitude is assumed to be dominated by scattering to other modes (population decay, i.e., energy relaxation) and not by pure dephasing from inhomogeneous ensemble effects.^[^ ^53^ ^]^ Therefore, half of the damping time extracted from the time‐domain fits is a reasonable upper limit for the phonon lifetime.^[^ ^54^, ^55^ ^]^ The temperature dependent phonon lifetimes for (4cat)PbBr_3_ are shown in Figure 4B. A shortening of the lifetime is observed with increasing temperature, from *τ_4cat,5K_* = 10.3 ps (±2.4) at 5 K to *τ_4cat,80K_* = 6.5 ps (±0.9) at 80 K, and until *τ_4cat,180K_* = 1.8 ps (±0.3) at 180 K. Moreover, the 1.1 THz mode exhibits a clear redshift with increasing temperature, as shown in Figure 4C. A comparison of the experimentally observed shift with the predicted shift due to lattice expansion and anharmonic phonon‐phonon scattering clearly shows that the lattice expansion term alone is insufficient to explain the observed shift in phonon frequency. Possible links between the observed optoelectronic properties, structure, and coherence time are discussed in the following section.

## 3 Discussion

### 3.1 Improved Morphology and Structural Phase Stability

Hydrogen bonds play a pivotal role in the structural stabilization of LHPs, and higher crystal phase stability can be explained by an increased number of H‐bonds with beneficial orientation within the lead‐halide framework.^[^ ^56^ ^]^ Compared to the MA*^+^* cation, GA*^+^* increases the number of interactions to six H‐bonds (Figure 1A) within the inorganic framework, significantly improving GA‐doped perovskite stability.^[^ ^57^, ^58^, ^59^ ^]^ Goldschmidt's tolerance factor *t*, defining the limits on ionic sizes for each component,^[^ ^60^ ^]^ is predicted to reach close to ideal value of *t* = 0.99 for the designed (4cat)PbBr_3_, whereas the cubic structure of MAPbBr_3_ only fulfills *t* = 0.92 (see Table S1, Supporting Information). This is experimentally confirmed by our thin film XRD results, revealing a cubic structure for both MAPbBr_3_ and (4cat)PbBr_3_ at room temperature (see Section S13, Supporting Information). The high symmetry of the space group *Pm* $\underset{-}{3}$ *m* is reflected in the observed diffraction pattern. Additionally, this space group was confirmed via the single crystal XRD at room temperature. However, the observed structural order and improved lattice coherence in the (4cat) system are not solely dependent on the tolerance factor. This is directly evident by the vanishing lattice coherence of (MA_0.2_FA_0.8_)PbBr_3_ in Figure 3a, despite having the same tolerance factor of t = 0.99. Achieving uniform, pinhole‐free, and smooth perovskite thin films becomes imperative to mitigate structural defects, ensuring the stable and enduring operation of optoelectronic devices.^[^ ^1^ ^]^ A lack of full film coverage results in inadequate light absorption, and creates shunt paths that can adversely impact the device efficiency.^[^ ^61^ ^]^ In this work, full film coverage with micro/nano grains (Figure 1B) has been optimized without using any additives via a one‐step spin‐coating approach. Surface and bulk defects possibly explain the lower PL emission of MAPbBr_3_ when compared to (4cat)PbBr_3_ thin films (Figure 2B), which show full coverage, uniform grain size distributions, and a smooth surface (Figure 1B right image).

To confirm GA⁺ presence, a triple cation GA⁺‐free perovskite was analyzed with High‐resolution X‐ray photoelectron spectroscopy (XPS) (Section S14, Supporting Information).^[^ ^62^ ^]^ The XPS spectrum (Figure S14B, Supporting Information) showed peaks for C 1s correlated to MA and FA and Cs 3d_5/2_. In contrast, the (4cat)PbBr₃ spectrum (Figure S14C, Supporting Information) displayed MA, FA, and GA peaks, confirming that GA alters chemical bonding, changing the local C 1s environment. The estimated surface composition by integrating the peak areas for the quadruple‐cation variant was (MA, FA, GA)_0.81_ Cs_0.03_PbBr_2.84_. Therefore, in comparison to the solution composition (GA_0.015_Cs_0.046_MA_0.152_FA_0.787_PbBr_3_) the organic cations exhibited a deviation of approximately 15%, while the Br/Pb ratio showed a deviation of around 5% from the stoichiometric composition of the precursor solution.^[^ ^63^ ^]^ Generally, the precise structure of multi‐cation perovskites remains a challenge, as seen from the long‐pending structural analysis of the most cited triple‐cation perovskite, which has been later provided in 2023.^[^ ^64^, ^65^ ^]^

Optical evaluation of thin films sheds light on how the composition affects the optoelectric properties of the compounds, via changes of ion size, PbX octahedral tilting, and the structural phase. The electronic structure is determined by the PbX inorganic sublattice (the band edges are built from hybridized metal and halide orbitals) and can remain largely unchanged with different A‐site cations.^[^ ^43^, ^66^ ^]^ Nevertheless, the different A‐site cations can still exert indirect influence through subtle interaction with the inorganic lattice. The absorbance data in Figure 2A (inset) indicate that the optical bandgap exhibits a red shift similar to the one observed for PL emission energy. The absorption onset occurs at higher energy for MAPbBr_3_ compared to (4cat)PbBr_3_ and at lower energy for FAPbBr_3_, in full agreement with literature reports on FA^+^ leading to a bandgap decrease, and MA^+^ to an increase.^[^ ^66^, ^67^ ^]^ The PL spectrum shows a peak at a longer wavelength (lower energy) compared to the absorbance edge, and such emission Stokes shift is typical for perovskite materials.^[^ ^68^ ^]^ Additionally, the observed single PL peak near the absorption edge in Figure 2B is indicative of a single crystallographic phase consistently observed across all investigated thin films, as supported by XRD results shown in Figure S13 (Supporting Information).^[^ ^69^ ^]^ PL emission becomes more intense for the mixed cation perovskites and the highest intensity is achieved for (4cat)PbBr_3_, and can be explained by the suppression of non‐radiative recombination in this perovskite layer. Additionally, this could be an indication of reduced trap density in the path of charge carriers and decreased photoluminescence quenching, which has a considerable impact on the photovoltaic performance of this sample.^[^ ^70^ ^]^ This can be supported by modified surface properties and a reduced defect density due to the introduction of GA^+^ ions into the perovskite lattice.^[^ ^45^, ^71^ ^]^ Our correlative impedance spectroscopy results in Figure 2C, demonstrating twenty times higher electron mobility (see Table S2, Supporting Information) for (4cat)PbBr_3_ (0.75 cm^2^ V^−1^ s^−1^) than MAPbBr_3_ (0.037 cm^2^ V^−1^ s^−1^), further supports our interpretation of reduced density of bulk defect sites (see Section S5, Supporting Information). Therefore, this study demonstrates the synergistic influence of A‐site engineering for enhanced electron mobility, resulting in a longer carrier diffusion length and superior optoelectronic performance in (4cat)PbBr_3_. Hence, despite the increased cation entropy that could potentially create more defect states, our observations confirm the suppression of non‐radiative recombination and electron mobility enhancement.^[^ ^70^ ^]^

Investigations on single crystal LHPs help to decipher the inherent characteristics of the pristine bulk material without the interference from morphological effects intrinsic to polycrystalline thin films discussed in the previous paragraph.^[^ ^38^ ^]^ LHPs undergo structural phase transitions from orthorhombic to tetragonal to eventually form the cubic phase with increasing temperature.^[^ ^72^ ^]^ Consequently, maintaining a cubic phase for 3D LHPs at room temperature or lower can decrease energy demands involved in annealing steps for device manufacturing. In addition, A‐site cation engineering was previously found to facilitate a negative formation enthalpy and enhanced thermodynamic stability.^[^ ^57^, ^73^ ^]^ Based on our results from temperature dependent XRD, we conclude that the (4cat)PbBr_3_ structure retains its symmetry over a wide temperature range and therefore exhibits a single crystal cubic phase down to at least 80 K. The observed lattice constant deviation is ≈ 0.1 Å and significantly smaller than the typical changes associated with phase transitions (e.g., for MAPbBr_3_ with ≈3.5 Å change of lattice constants) and changes happen slowly and continuously in the entire measured temperature range, unlike in the case of a distinct phase transition.^[^ ^74^ ^]^ The Raman spectra in Figure S11C (Supporting Information) show no peak splitting or abrupt frequency changes of higher frequency modes with decreasing temperature, further supporting high structural order without any phase transition down to 80 K.^[^ ^75^, ^76^ ^]^ A previous work by Zeiske et al. showed that the static disorder, manifested in the Urbach energy *E*_u_, decreases with an increasing number of cations at cryogenic temperature and might indicate an improved structural order.^[^ ^77^ ^]^ Our work fully supports this notion, by unveiling that even higher number of cations improve the structural order of the material at 80 K, which could explain the longer phonon coherence time observed in (4cat)PbBr_3_ compared to MAPbBr_3_.

### 3.2 Extended Lattice Coherence Times

Despite the expected variations of lattice distortions when mixing different sizes of cations in one compound, we strikingly find the dominating vibrational mode of the inorganic lattice, which strongly couples to the nonlinear THz polarizability, to remain the same in (4cat)PbBr_3_ as in MAPbBr_3_.^[^ ^20^ ^]^ At 80 K, the mode displays a doubled coherence time compared to the pure MAPbBr_3_ compound. Notably, this is not the case for all investigated thin films, but special to the specific (4cat)PbBr_3_ composition (see Figure 3A). Therefore, if Cs^+^ or GA^+^ were not incorporated in the thin film, we would not have observed this evidently recovered coherent response compared to FAPbBr_3_ and (FA, MA)PbBr_3_. Our TKE results thus provide a strong fingerprint of a changed structural composition. As the observed 1.1 THz octahedral tilting mode not only governs the structural phase transition but may also contribute to charge‐screening effects, ^[^ ^20^ ^]^ these findings suggest a link between the improved optoelectronic properties and enhanced lattice coherences, which could be a signature of lower defects. In Figure 3A,B, we observe significant differences in phonon lifetimes between (MA, FA)PbBr_3_, pure FAPbBr_3_, and (4cat)PbBr_3_. Surprisingly, (4cat)PbBr_3_, despite its substantial 79% FA^+^ content at the A‐site, exhibits remarkably long‐lived phonon modes. Despite the minimal presence of MA, the same 1.1 THz mode as in pure MAPbBr_3_ dominates the coherent lattice response.^[^ ^20^ ^]^ Notably, while the 1.1 THz mode is clearly present in both MAPbBr_3_ and (4cat)PbBr_3_, it is absent in (FA_0.8_MA_0.2_)PbBr_3_. This observation prompts us to consider the potential role of GA^+^ and Cs^+^ in reinforcing the dynamics of the inorganic lattice of the benchmark MAPbBr_3_ compound. To our knowledge, the coupling of the lattice to the GA^+^ cation and its impact on the mixed‐cation LHPs have not been thoroughly elucidated so far, although it has been proposed recently that the steric hindrance created by the large GA^+^ cations distorts and rigidifies the inorganic Pb‐X network.^[^ ^78^ ^]^ Our observations lead to the hypothesis, that the chemical composition of FA^+^, GA^+^, and Cs^+^ plays a pivotal role in stabilizing the overall lattice behavior, despite the variations in cation size. This suggests a complex interplay between A‐site cations and their influence on the static structure and cage dynamics, opening avenues for further investigation, toward better understanding and optimization. (4cat)PbBr_3_ additionally exhibits few short‐lived vibration modes between 2.2 THz and 3.3 THz, which might be additional fingerprints of the increased coherence time (see Section S15, Supporting Information). Moreover, the most prominent short‐lived mode at 73 cm^−1^ (2.2 THz) coincides with the dominating Raman feature in Figure S11C (Supporting Information).

These observations cannot be explained by phase segregation phenomena, which are particularly prevalent in mixed‐cation LHPs^[^ ^79^ ^]^ and have been reported to impact optoelectronic properties.^[^ ^80^ ^]^ For a probed region with locally enhanced MA^+^ presence, it would be reasonable to anticipate a more pronounced response governed by the 1.1 THz mode at 80 K. However, it is important to consider that our TKE experimental geometry, with a probe focus diameter of ≈50 µm and grain dimensions of hundred nanometers, averages out these effects of segregated phases.^[^ ^45^ ^]^ The reproducibility of different sample areas and at different times provides additional evidence that the observed 1.1 THz mode is an inherent response of the specific (4cat)PbBr_3_ mixing ratio (Section S9, Supporting Information). Coming back to the work of Zeiske et al., which unveiled that the Urbach energy *E*_U_ decreases with an increasing number of incorporated cations, ^[^ ^77^ ^]^ the dynamic lattice response further supports the improved structural order in such systems. The intricate mixture of (4cat)PbBr_3_ might unexpectedly contribute to an elevated level of structural ordering. Although it appears counterintuitive to observe a prolonged phonon coherence in a material with more heterogeneous cation composition, the dynamics of the inorganic sublattice may benefit from a globally higher structural order in the presence of a diverse cation mixture.

### 3.3 Anharmonic Phonon Scattering

In the last step of this study, we aim to understand the temperature evolution of the phonon coherence by modeling the anharmonic phonon‐phonon decay. The main decay channel of the observed optical phonons at the center of the Brillouin zone is phonon‐phonon interaction due to intrinsic lattice anharmonicity, scattering to two or more lower‐energy phonons with higher momenta. The temperature dependence of the linewidth *Γ =* 1/(2πτ), where τ is the phonon lifetime, is fitted by a simple model assuming only the decay of the initial optical phonon into two (cubic anharmonicity, Equation S16 and Section S16, Supporting Information) or three (quartic anharmonicity, Equation S17 and Section S16, Supporting Information) equally energetic phonons and a temperature‐independent impurity scattering term Γ_0_ (see details in Experimental Section).^[^ ^81^, ^82^ ^]^ As seen in Figure 4B,C, both models capture the overall trend of lifetime decrease with temperature, but deviate from the experimental data in the 5–80 K range, which suggests more complex decay channels at low temperatures. Given the lack of precise phonon dispersion data, we refrain from assigning specific decay channels. Nevertheless, here, we can already conclude that the observed decoherence is consistent with an anharmonic decay and not dominated by inhomogeneous broadening effects.

Phonon frequency shifts with temperature are a consequence of lattice anharmonicity, which is manifested in the effects of crystal thermal expansion and renormalization of self‐energy due to anharmonic phonon‐phonon interaction (see Section S17, Supporting Information). The temperature‐dependent phonon frequency can thus be expressed as a sum of those contributions:

(1) $$\omega \left(T\right)=\omega_{0}+\Delta_{0}\left(T\right)+\Delta_{anh}\left(T\right)$$

where ω_0_ is the extrapolated harmonic frequency of the mode at *T* = 0 K, Δ_0_(*T*) is the shift due to thermal lattice expansion, and Δ_*anh*_(*T*) is the shift due to anharmonic phonon‐phonon coupling. We estimate Δ_0_(*T*) based on the lattice expansion coefficient extracted from our XRD data and consequently fit the remaining shift Δ_*anh*_(*T*) by simple models considering symmetric phonon decay due to cubic or quartic anharmonicity (see Section S16, Supporting Information). Consistent with our observation for the phonon lifetimes, the discrepancy between the simple model predictions and the experimental results in the 5–80 K range suggest more complex mechanisms at play in the lower temperature range.

### 3.4 Impact on Dynamic Screening Models

The observation of a longer phonon coherence time in a material with higher A‐site cation complexity seems at the first instance counterintuitive, however considering the improved charge carrier mobility and structural ordering in the inorganic sub‐lattice it appears plausible. Previously, it has been suggested that 1.1 THz octahedral twist mode may nonlinearly contribute to charge‐screening effects in MAPbBr_3_.^[^ ^20^ ^]^ In this work, we find that the same mode is observed in (4cat)PbBr_3_, but with even increased lattice coherence time that may help to build up the initial dynamic screening mechanism. The concept of carrier screening has been widely discussed in the perovskite community as a potential explanation for the exceptional properties of LHPs.^[^ ^24^, ^83^, ^84^ ^]^ Here, we propose that longer lattice coherence supports dynamic charge screening of the charge carriers. This could offer an additional explanation for the reduction of non‐radiative recombination observed via photoluminescence enhancement, complementing the improved passivation of grain boundaries. However, this hypothesis requires further in‐depth investigations, particularly to assess the strength of electron‐phonon coupling in (4cat)PbBr_3_. Our experimental results do not provide full clarity on this matter, prompting further studies on the intricate interplay of screening effects in (4cat)PbBr_3_. Complementary to indirect studies involving interpretations of the Urbach tail, the TKE directly follows the more concerted motion of the lattice, most likely arising from a globally improved structural order. Our findings motivate future investigation of the Urbach energy in (4cat)PbBr_3_, particularly at cryogenic temperatures, and play a pivotal role in shaping the landscape of high‐impact optoelectronic technologies with a deeper understanding of lattice dynamics.

## 4 Conclusion

This work on quadruple A‐site cation LHPs provides intriguing insights into the subtle and intricate interplay of microstructure, crystal structure, optoelectronic properties, and ultrafast lattice dynamics by studying the respective single crystals and thin films. One of the major findings here is that incorporating GA^+^ cations in the quadruple A‐site cation lead bromide perovskite leads to higher structural stability in the cubic phase, increased PL efficiency, improved charge mobility, and extended vibrational lifetime of the inorganic lattice, defying the conventional expectations. By pioneering TKE measurements on multiple A‐site cation LHPs, we demonstrate that (4cat)PbBr_3_ perovskite displays a more than doubled phonon coherence time compared to the MAPbBr_3_ parent compound. This indicates that the ultrafast inorganic lattice response behaves in a more synchronized manner despite the more diverse cation mixing in quadruply substituted perovskites. Our counterintuitive findings support previous works, which introduced the concept of enhanced structural order by an increased number of cations.^[^ ^77^ ^]^ The presence of multiple cations in (4cat)PbBr_3_ thus seems to enforce structural order at 80 K and below, while extending the phonon lifetime, but not altering the dominating lattice frequency compared to MAPbBr_3_. Our findings further indicate that (4cat)PbBr_3_ facilitates the built‐up of dynamic charge‐carrier screening, which would contribute to its higher diffusion coefficients and increased PL yield. Therefore, despite an increased structural complexity due to the varying A‐site cation size, our specific (GA_0.015_Cs_0.046_ MA_0.152_FA_0.787_)PbBr_3_ composition shows great promise in shaping the landscape of future optoelectronic materials and will stimulate further high‐impact investigations of this compound.

## 5 Experimental Section

### Absorption and Photoluminescence (PL) Setup

The absorption setup was Lambda 1050 and PL setup was LS 55, both from Perkin Elmer. The absorption spectrometer was a Tungsten Halogen as well as a Deuterium lamp that were used as a light source and the absorbance was measured by using a reference substrate sample. For the PL spectrometer a Xe‐lamp was used with a monochromator resulting in 10 nm wide “monochromatic” light. The excitation light was filtered by utilizing a vertical linear polarizer prior to excitation, while the outcoming PL was filtered using a horizontal linear polarizer. This geometry can reduce the scattering contribution.

### Electrochemical Impedance Spectroscopy (EIS)

Measurements were conducted on a Versa‐STAT 4 Potentiostat/Galvanostat/Frequency Response analyzer (Princeton Applied Research, Ametek Inc.). A 10‐mV amplitude of the input potential perturbation was used to measure the potentiostatic impedance at the open circuit voltage (OCV) in the frequency range of 1 MHz to 100 mHz.

### Phonon Lifetime Extraction

Phonon lifetime *τ* is related to the linewidth Γ in frequency domain *τ = (2πΓ)* ^−1^. Alternatively, the exponential damping constant ζ in time domain corresponds to ζ *= πΓ*. We base the analysis on the damping constant ζ extracted from fits in time domain to avoid additional broadening artifacts of the peak in frequency domain due to the limited measurement temporal window.

### Raman Setup

Raman spectra were obtained using a Renishaw InVia Qontor spectrometer equipped with a 785 nm triple diode laser delivering 100 mW output energy. The measurements were conducted in 180 °C backscatter geometry with 1800 lines mm^−1^ grating, a 65 µm slit opening, and a Rayleigh edge filter with a cut‐off below ≈50 cm^−1^. A front‐illuminated CCD Centrus detector captured the data, achieving an estimated spectral resolution of about 0.4 cm^−1^. For temperature control, a Linkam THMS600 stage was used, in which the single crystal was situated and measured with a x50 LWD objective (NA = 0.5) through the top quartz glass window. Temperature steps were manually set, ranging from room temperature to the limit of nitrogen cooling (80 K). Spectral acquisition occurred after allowing the system to stabilize for several minutes at each temperature step. Before data collection, the laser focus was meticulously readjusted to the same sample location to compensate for minor shrinkage and movement induced by the cooling process. Spectra were recorded using a transmission filter of 10% over 10 times 1 s.

### Scanning Electron Microscopy (SEM)

Surface morphology images have been acquired with a Zeiss Sigma 300 VP instrument.

### Thin Film Fabrication

Glass substrates, including BK7 glass and microscopic slides, underwent a cleaning process. Initially, they were treated with a 2% Hellmanex water solution and subjected to 15 min of sonication. Following this, they underwent an additional 15‐minute sonication process in acetone followed by isopropyl alcohol. The cleaning process terminated with drying using a nitrogen flow. Subsequently, a 15‐minute treatment in a UV‐ozone chamber before the deposition of the perovskite layer was done. The perovskite compositions namely, MAPbBr_3_, CsPbBr_3_, FAPbBr_3_, and (FA_0.8_MA_0.2_)PbBr_3_, (GA_0.015_Cs_0.046_ MA_0.152_FA_0.787_)PbBr_3_ shorten to(4cat)PbBr_3_ were prepared by mixing specific amounts of powder precursors in DMF and DMSO solvents with a ratio of 80:20 Vol%. These solutions were applied using a one‐step spin coating method, with 200 µL of chlorobenzene added in the final 5 s of spinning as an antisolvent. The resulting layers were annealed at 100 °C for 1 h within a nitrogen‐filled glove box. For each composition, an optimized program was utilized, ensuring precise control over the thickness of the films.

### THz‐Induced Kerr Effect (TKE)

Optical rectification in LiNbO_3_ using the tilted pulse front technique was used to generate THz pulses, ^[^ ^85^ ^]^ with a central frequency of 1.0 THz and a field strength of 1.5 MV cm^−1^. To achieve this, LiNbO_3_ was excited by laser pulses from an amplified Ti:sapphire laser system (central wavelength, 800 nm; pulse duration, 35 fs FWHM; pulse energy, 5 mJ; repetition rate, 1 kHz). The probe pulses were synchronized with the THz pulses and originated from a Ti:sapphire oscillator (central wavelength, 800 nm; repetition rate, 80 MHz), being collinearly aligned and temporally delayed relative to the THz pulse, and set at 45° with respect to the vertically polarized THz pulses. The latter induced a change in birefringence known as the Terahertz‐Induced Kerr Effect within the sample.^[^ ^86^ ^]^ This birefringence results in the probe field acquiring a phase difference between polarization components parallel and perpendicular to the polarization of the THz pulse. The phase difference was detected using a combination of a half‐wave and quarter‐wave plate, going along with a Wollaston prism in order to spatially separate perpendicularly polarized probe beam components.^[^ ^20^ ^]^ Finally, two photodiodes in a balanced detection configuration were used to detect the two separated beams’ intensity.

### X‐Ray Diffraction (XRD)

The polycrystalline thin films XRD analysis was done using a STOE‐STADI MP diffractometer manufactured by STOE. X‐ray beam generated with *Cu Kα* (*λ* = 1.5406 *Å*) source and operated at 40 kV and 40 mA in reflection mode. The single crystal X‐ray diffraction (SC XRD) analysis was conducted using a Bruker D8 Venture instrument with Mo radiation (*λ* = 0.71073 *Å*), operating at 50 kV and 1.4 mA. The collected frames were integrated using the Bruker SAINT software package with a narrow‐frame algorithm.

## Conflict of Interest

The authors declare no conflict of interest.

## Supporting information



Supporting Information

## Data Availability

The data that support the findings of this study are available from the corresponding author upon reasonable request.
