# Supplementary material for: Enhanced Lattice Coherences and Improved Structural Stability in Quadruple A‐Site Substituted Lead Bromide Perovskites
Source: Small. 2025 Apr 18;21(21):2500977. doi: 10.1002/smll.202500977 (PMC12105430; doi:10.1002/smll.202500977)
Supplement: Supplementary file 1 — Supporting Information [file SMLL-21-2500977-s001.docx]

Supporting Information

**Enhanced Lattice Coherences and Improved Structural Stability in Quadruple A-Site Substituted Lead Bromide Perovskites**

Marie Cherasse, Niusha Heshmati, Joanna M. Urban, Feray Ünlü, Michael S. Spencer, Maximilian Frenzel, Luca Perfetti, Sanjay Mathur*, Sebastian F. Maehrlein*

**Section S1. Sample preparation methods**

**(4cat)PbBr_3_ Single crystal growth:** The inverse temperature crystallization (ITC) method is used to grow single crystals.^[1]^This method leverages the unique solubility behavior of perovskite precursors, where the solubility decreases with increasing temperature, contrary to most materials.

To begin the process, a precursor solution is prepared by dissolving (2 mol) of PbBr₂ along with (0.03 mol) of GABr, (0.092 mol) of CsBr, (0.304 mol) of MABr, and (1.574 mol) of FABr, in a high-boiling-point solvent system in a nitrogen filled glovebox. Our optimized solvent system is N,N-dimethylformamide (DMF): γ-butyrolactone (GBL): dimethyl sulfoxide (DMSO) with (80:10:10 vol%). The final 1 (_M_) solution was heated at 60 °C for one hour and then the temperature was increased to 150 °C and kept for 3 minutes. Then the solution was cooled down to 50 °C resulting in a clear and colorless supersaturated solution, which was filtered through a 0.22 μm PTFE syringe filter. The filtered solution was transferred to a programmable oven, where the temperature was initially set at 60 °C. The temperature was then gradually increased by 5 °C per hour. Crystal seeds began to form at 90 °C, after which the temperature was raised by 10 °C per day to promote the growth of larger crystals. By the time the temperature reached 120 °C, large crystals had developed. The crystals were then removed from the solution by carefully decanting the remaining liquid and gently rinsing them with isopropanol. Finally, the crystals were dried and stored under a nitrogen atmosphere to prevent degradation.


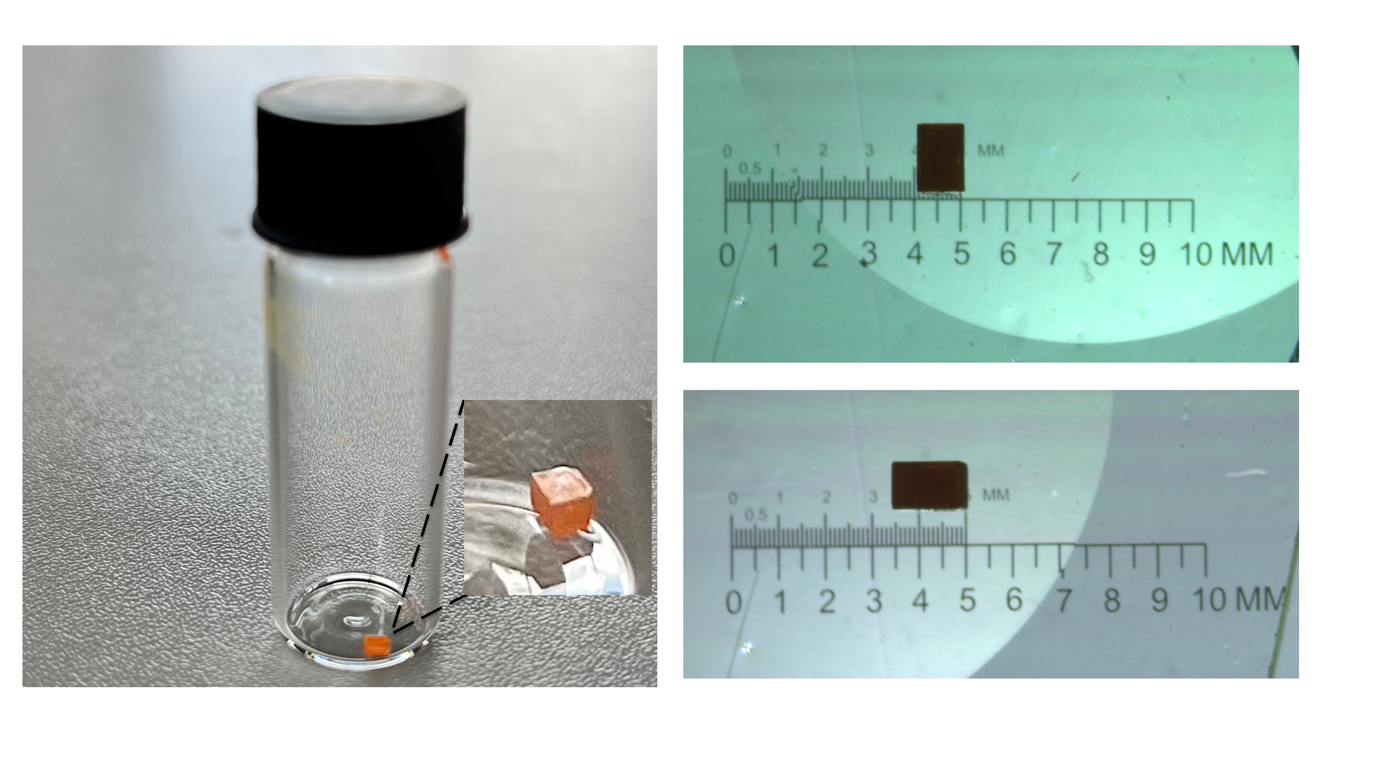


**Figure S1.** The GA_0.015_Cs_0.046_MA_0.152_FA_0.787_PbBr_3_ single crystal growth via inverse temperature crystallization (ITC) method and results in high quality crystal with size of 1$\times$1.5 mm^2^.

**(4cat)PbBr_3_ Thin film fabrication:** For the preparation of a (4cat)PbBr_3_ perovskite solution, (0.03 mol) of GABr, (0.092 mol) of CsBr, (0.304 mol) of MABr, and (1.574 mol) of FABr as our cation salts and (2 mol) of PbBr₂ was added to complete the precursor salts in N_2_ atmosphere glovebox. The optimum solution concentration is 1(_M_) and prepared by adding DMF:DMSO (80:20 vol%). The solution was heated at 60 °C for one hour, resulting in a clear, colorless solution, which was subsequently filtered using a 0.22 μm PTFE syringe filter. The perovskite layer was deposited on the substrate via a one-step static spin coating process, where the solution was spun at 1000 rpm for 10 seconds followed by 5000 rpm for 40 seconds. Five seconds before the end of the spin coating, (250 μL) of chlorobenzene was introduced as an anti-solvent. Finally, the substrate/perovskite sample was annealed at 100 °C for 60 minutes to achieve highly crystalline perovskite thin film. The final perovskite film achieved a thickness of $\approx$350-370 nm (see Figure S2). The same process, including the carefully optimized concentration of the precursor solution and the specific spin coating parameters, was applied for all thin films to ensure a consistent thickness for each sample.


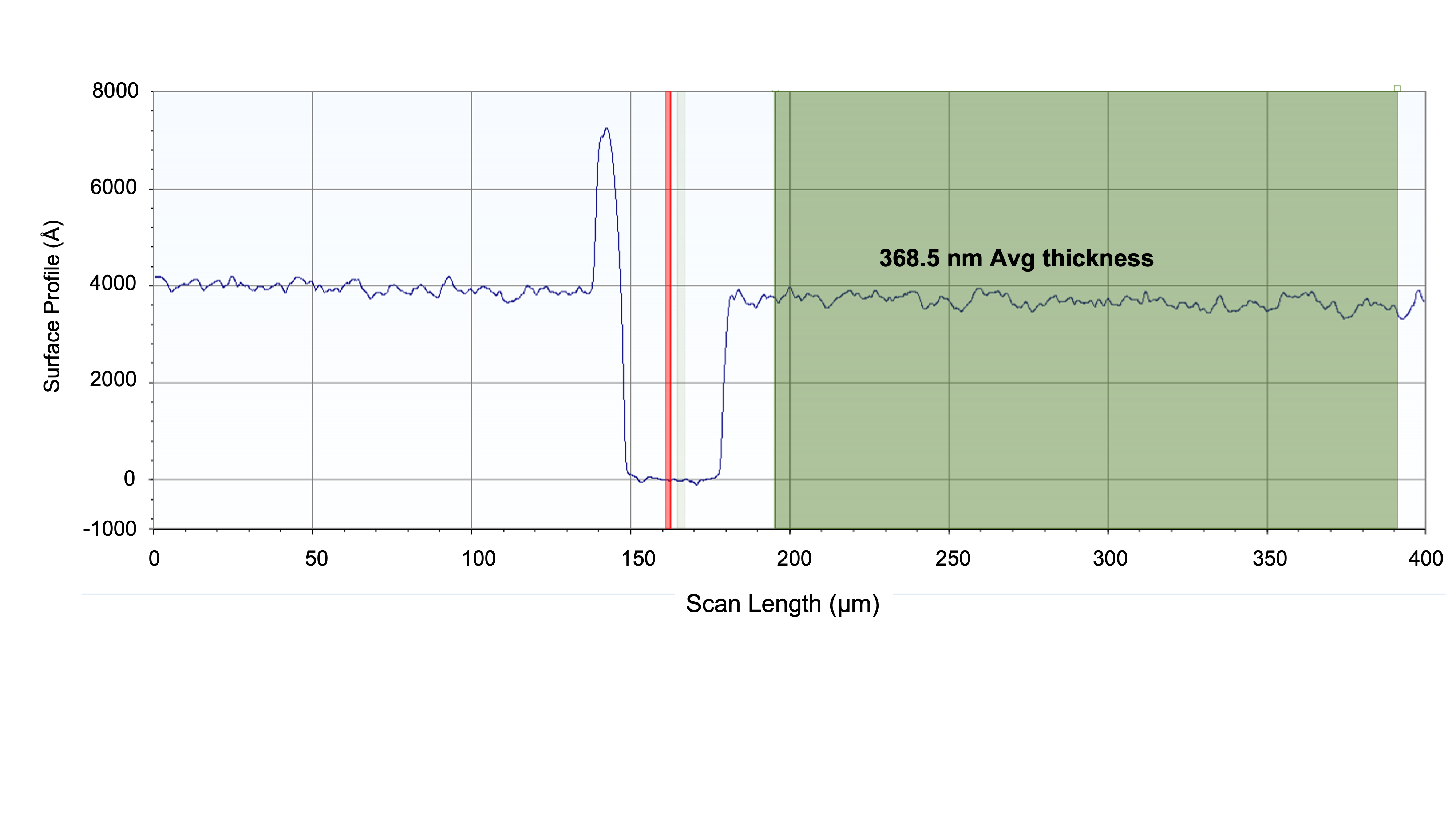


**Figure S2.** Thickness assessment of the (4cat)PbBr_3_ film using a Dektak profilometer, revealing an average thickness of 368.5 nm.

**Section S2. Tolerance factor**

The crystallographic stability and the probable crystal structure of halide perovskite can be predicted by the Goldschmidt’s tolerance factor (*t*), which is defined by the following formulas (Equation S1) and R_A_, R_B,_ and R_X_ are the ionic radii of A, B cations and X anion, respectively. The tolerance factor describes the ratio of distances between A—X and B—X regarding their ionic radii under the boundary condition of a solid sphere. If the calculated results are in the range of 0.81<*t*<1.11, this is favorable to the formation of a 3D-crystal structure. It is reported that an ideal cubic structure will be preferably formed when the value of *t* locates at a narrower range of 0.89–1.0, whereas a less symmetric tetragonal or orthorhombic structure may be present if the value of *t* is lower than this range. In general, Goldschmidt´s tolerance factor was introduced to define the deviation from the ideal cubic crystal structure *t*=1.^[2,3]^

$t=\frac{R_{A}+R_{X}}{\surd2(R_{B}+R_{X})}$ *(S1)*

**Table S1**: Calculated t values for different perovskite compositions.

| Perovskite | Tolerance factor (*t*) | Ionic Radii of A (*R_A_*) [pm] |
| --- | --- | --- |
| CsPbBr_3_ | 0.86 | 188.0 |
| MAPbBr_3_ | 0.92 | 217 |
| FAPbBr_3_ | 1.01 | 253 |
| GAPbBr_3_ | 1.06 | 278 |
| MA_0.2_FA_0.8_PbBr_3_ | 0.99 | 245.5 |
| (4cat)PbBr_3_ | 0.99 | 244.9 |

**Section S3. Surface morphology**

To investigate the surface structure, thin films with a 350 nm thick photoactive layer were analyzed via scanning electron microscopy (SEM). Figure S3 shows the FAPbBr_3_ and (MA_0.2_FA_0.8_)PbBr_3_ thin films under different magnifications. In the case of FAPbBr_3_ fabricating a high-quality film with full coverage is a demanding task, and it becomes more challenging with one-step deposition methods that was utilized to make these films.^[4,5]^ For double cation perovskites, A-site engineering improved film quality and achieved full coverage, eliminating all the small holes in the FA perovskite. Additionally, the unreacted PbBr_2_ phase visible in FAPbBr_3_ (needle shape in grain boundaries), resulting from incomplete reaction and conversion to perovskite, was removed in double cation perovskite.

**
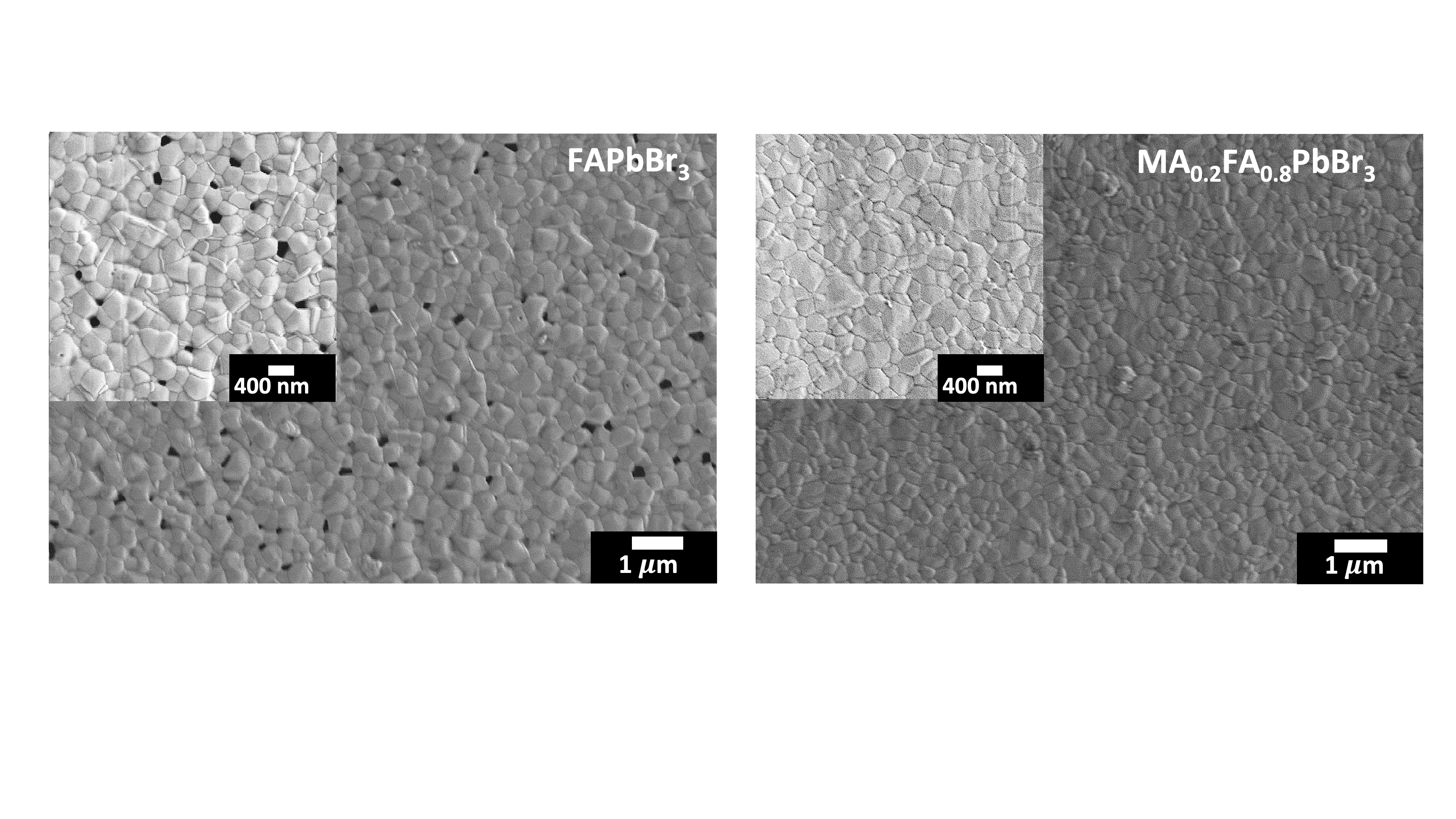
Figure S3.** SEM images of fabricated FAPbBr_3_ and MA_0.2_FA_0.8_PbBr_3_ thin films at room temperature and one step spin coating method.

**Section S4. Urbach energy approximation**

In this study, the Urbach energy (*E_U_*) was calculated using UV-Vis absorbance data. The absorption coefficient (*α*) was determined from the relationship:

$\alpha=\frac{2.303\times A}{d}$ *(S2)*

Where *A* is the absorbance and *d* is the thickness of the film. To obtain the Urbach energy, a plot of $\ln\left( \alpha\right)$versus photon energy (*E*) was made, focusing on the linear region corresponding to the Urbach tail. The equation governing this region is:

$\ln\left( \alpha\right)=\frac{E}{E_{U}}+C$ *(S3)*

The inverse of the slope of the linear fit gives the Urbach energy:

$E_{U}={(\frac{dln()}{dE})}^{-1}$ *(S4)*

It is important to note that this method provides only an approximate value of the Urbach energy, taking into account the inherent uncertainties in both the measurement and fitting processes.


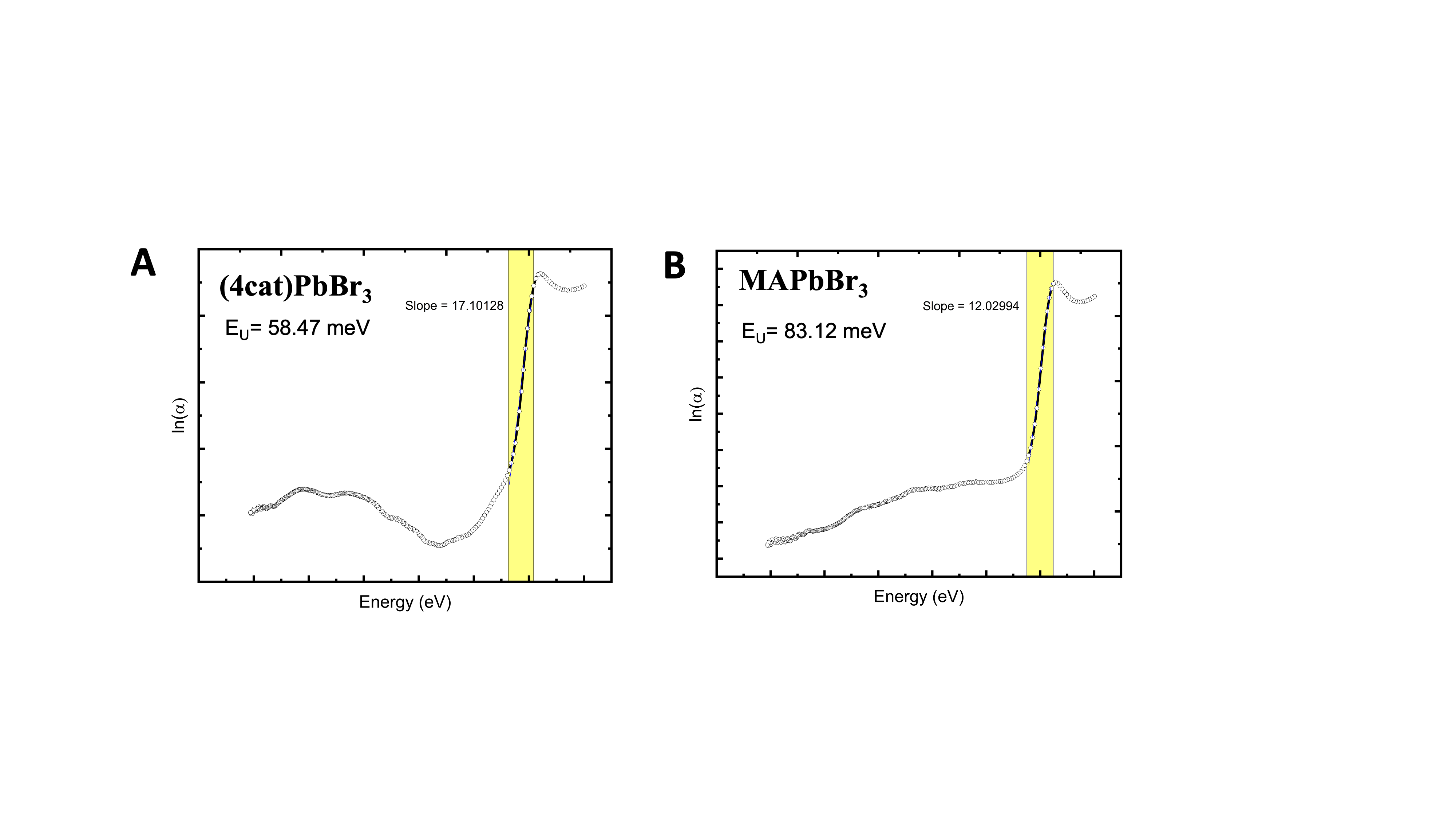


**Figure S4.** Determination of Urbach energy through two methods: (A, B) Absorption coefficient (α) derived from UV-Vis spectra, where the inverse slope of the linear region indicates the Urbach energy for (A) (4cat)PbBr₃ and (B) MAPbBr₃. (C, D) Urbach energy calculated from the PL spectrum by dividing the emission by the black body spectrum, showing the Urbach tail for (C) (4cat)PbBr₃ and (D) MAPbBr₃.

**Section S5. Diffusion mobility with Nernst-Einstein relationship**

Impedance spectroscopy (IS) is a well-stablished technique which can be utilized to identify the dynamic electrical transport properties that are occurring within the perovskite thin film.^[8]^ Each impedance arch can be considered by one RC element in an electrical equivalent circuit (EEC) model as shown in Figure S5 and represents a single RC element. In this work, devices were prepared with FTO/Perovskite/Au structure and perovskite were deposited on FTO and no charge selective material was used, therefore the Nyquist plots display a single arch which can attributed to the bulk recombination resistance and geometrical capacitance.^[9,10]^


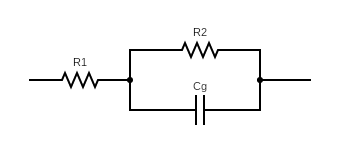


**Figure S5.** Electrical equivalent circuit (EEC) model.

There are successful models for calculating the diffusion coefficient. These models consider the diffusion length equal to the thickness of thin film electrodes and *τ_d_* is a diffusion transit time results from 45° Warburg element.^[11]^ Based on the Nyquist plot for a single-layer perovskite setup without electron transport and hole transport layers, the observation of the 45° Warburg element is rarely possible due to its overlap with other influencing factors.^[11]^ Therefore, considering the loss tangent (*tan δ*) of a capacitor:^[12]^

$\varepsilon^{'}= \frac{Z"}{\omega C_{0}({Z^{'}}^{2}+{Z^{"}}^{2})}$ *(S6)*

$\varepsilon^{"}= \frac{Z'}{\omega C_{0}({Z^{'}}^{2}+{Z^{"}}^{2})}$ *(S7)*

$\tan\delta=\frac{\varepsilon^{"}}{\varepsilon^{'}}= \frac{Z'}{Z"} (loss tag)$ *(S8)*

The relaxation time τ_2_ was calculated from the frequency corresponding to the maximum in the loss tangent versus frequency plot, assuming the model applicable for lead halide perovskites.^[13]^

The diffusion mobility can be calculated by applying the Nernst–Einstein relationship (equation S9), where *μ_e_* is diffusion mobility, e is electronic charge, *D* is chemical diffusion coefficient (equation S10), and *K_B_T* is the thermal energy at absolute temperature.^[14]^

$\mu_{e}=\frac{eD}{K_{B}T}$ *(S9)*

$D=\frac{L^{2}}{\tau_{2}}$ *(S10)*

$\tau_{i}=\frac{1}{2\pi f_{i}}$ *(S11)*

Here, we considered *L* equal to the perovskite layer thickness 350 nm. Hence, according to mentioned equations electron mobility was calculated and brought in Table S2.

**Table S2:** Calculated diffusion mobility by applying the Nernst–Einstein relationship.

| Perovskite | Thickness (*L*)  [nm] | Diffusion time (*τ_2_*_)_ [μs] | Diffusion coefficient (*D*) [cm^2^ s^-1^] | Electron mobility (*μ_e_*) [cm^2^ V^-1^ s^-1^] |
| --- | --- | --- | --- | --- |
| MAPbBr_3_ | 350 | 1.26 | 0.97×10^-3^ | **3.7×10^-2^** |
| (4cat)PbBr_3_ | 350 | 0.06 | 19.3×10^-3^ | **0.75** |

**Section S6. THz-induced transient birefringence temporal domain for different A-site thin films**


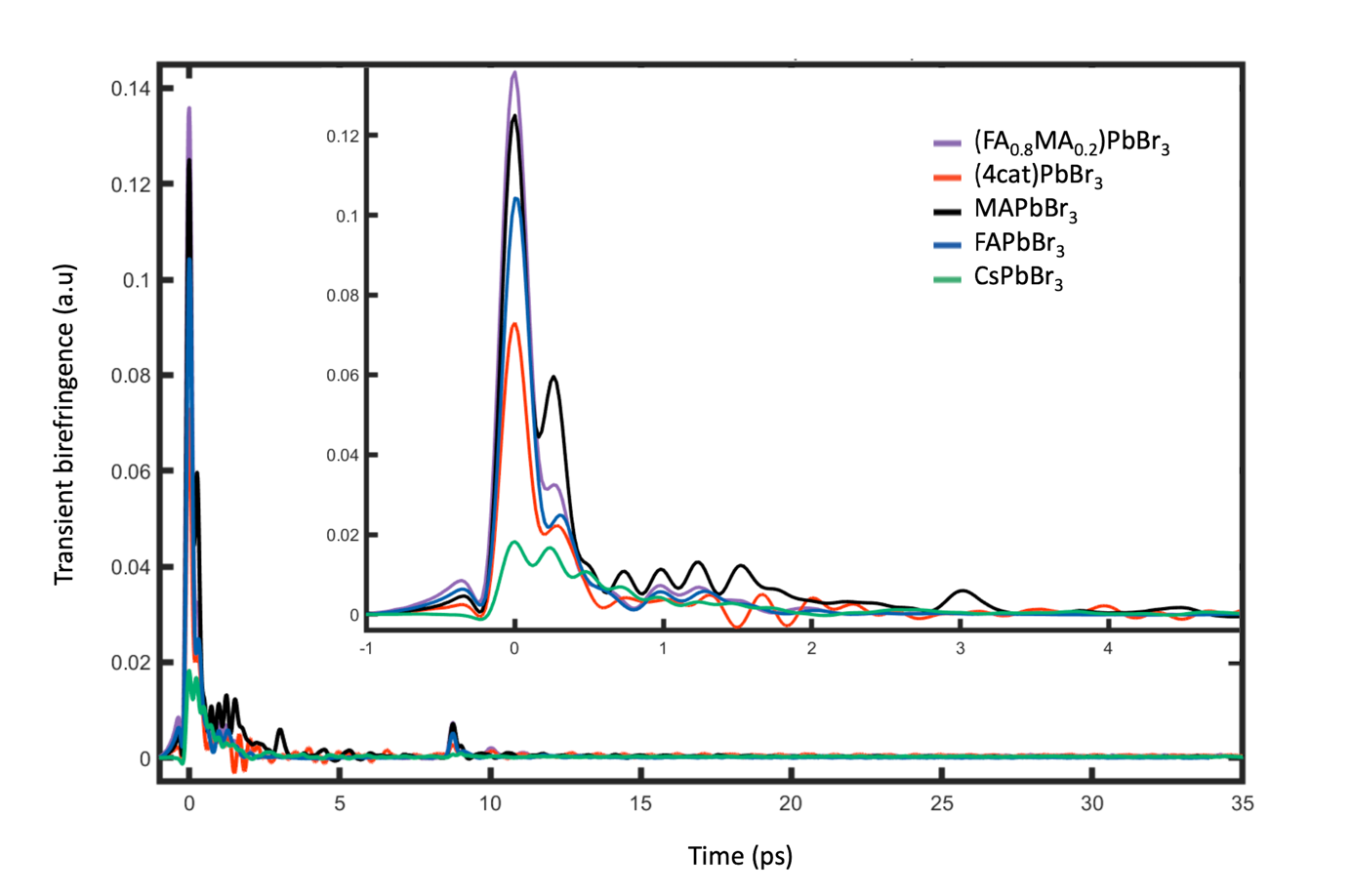


**Figure S6. Full THz-induced transient birefringence temporal domain for different A-site thin films:** Full THz-induced transient birefringence in different A-site cation lead bromide perovskites 350 nm thin films at 80 K, with a zoom on the first picosecond (box). The peak around 8 ps is attributed to the beam's reflection from the diamond windows of the cryostat and is visible in all the samples.

(4cat)PbBr_3_, MAPbBr_3_, (FA_0.8_MA_0.2_)PbBr_3_, and FAPbBr_3_ exhibit short-lived instantaneous electronic polarizability α_e_ ^[15]^, while CsPbBr_3_ thin film displays a more prolonged exponential tail of approximately 2 ps (Figure S6), as previously observed.^[12]^ We can further notice that all thin films manifest small-amplitude oscillations between 1 to 2 ps, with a 4 THz frequency. These oscillations are consistent across all thin films and are not attributable to the BK7 substrate, as evidenced by the negligible BK7 signal compared to the TKE signal generated by the LHPs thin film (Figure S7). Additionally, BK7 signal remains largely unchanged from room temperature to 80 K, further validating this argument.


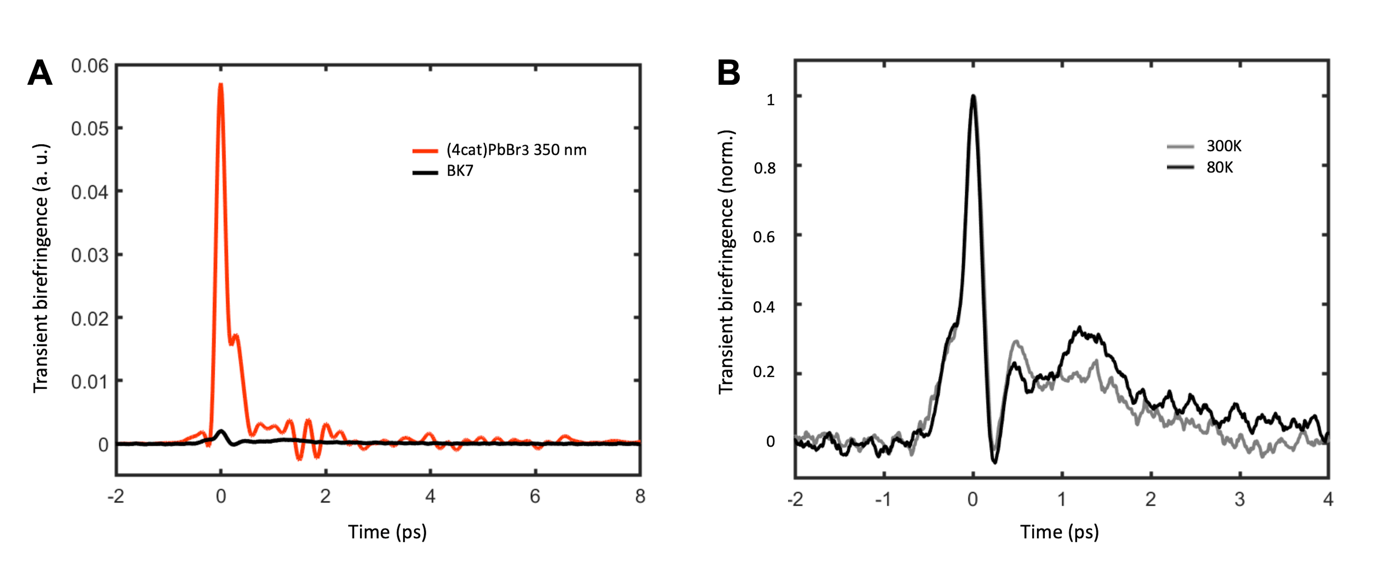


**Figure S7. BK7 substrate** A) Comparison of THz induced Kerr signal at 80K between BK7 substrate (black) and (4cat)PbBr3 350 nm thick thin film (orange). (B) THz induced Kerr signal in 500 µm BK7 glass substrate, at 300 K (grey) and 80 K (black). We conclude BK7 does not affect the quadruple cation LHPs signal.

Short-lived oscillations observed in FAPbBr_3_ in Figure 3A and Figure S6 are similar to Raman modes observed in former works.^[16,17]^ Nevertheless, the TKE FT does not show a broad THz mode at 1.1-1.4 THz as observed in Raman spectroscopy measurements.^[17]^ This may be connected to different dynamics of the FA cation and its coupling to the perovskite lattice, in comparison to the MA cation^[18]^ NMR measurements have indicated that FA-based compounds possess broader optical phonon modes compared to MA-based counterparts, attributed to the faster reorientation of FA within the lattice, despite its larger size.^[19]^ Even though the faster FA reorientation dynamics were suggested to improve the optoelectronic properties by enhanced charge carrier screening effects,^[20]^ our findings indicate that they are detrimental to long-lived lattice coherences.

**Section S7. Oscillatory signal fitting in time domain**

The oscillatory part of the transient birefringence signal was extracted from the TKE traces in the 5-180 K range by subtracting the non-oscillatory background originating from the instantaneous electronic response and propagation effects and fit with a single oscillator model for *t* > *t_cut-off_* to extract coherence times and exact frequencies of the 1.1 THz mode,^[15]^ as explained in the following paragraph.

The TKE data after subtracting the incoherent tail and cut to *t*> *t_cut-off_* was fit with an exponentially damped sinusoidal function according to the equation:

$S\left( t \right)=A\cdot\exp\left( -\zeta t \right)\cdot\sin\left( 2\pi ft+\varphi\right)+B$*(S12)*

The amplitude decay time *τ_A_* = $\zeta^{-1}$ corresponds to twice the phonon lifetime τ (assuming the absence of elastic scattering leading to pure dephasing), *τ_A_* =2τ.

We used *t_cut-off_* =5 ps for data in the 145-180 K range (where the modes are shorter lived) and *t_cut-off_* =9 ps for data in the 5-80 K range (where at short times contributions from the higher-frequency modes hinder the analysis of the 1.1 THz mode oscillatory signal). Exemplary fits for (4cat)PbBr_3_ and MAPbBr_3_ at 80 K are shown in Figure S8.

*
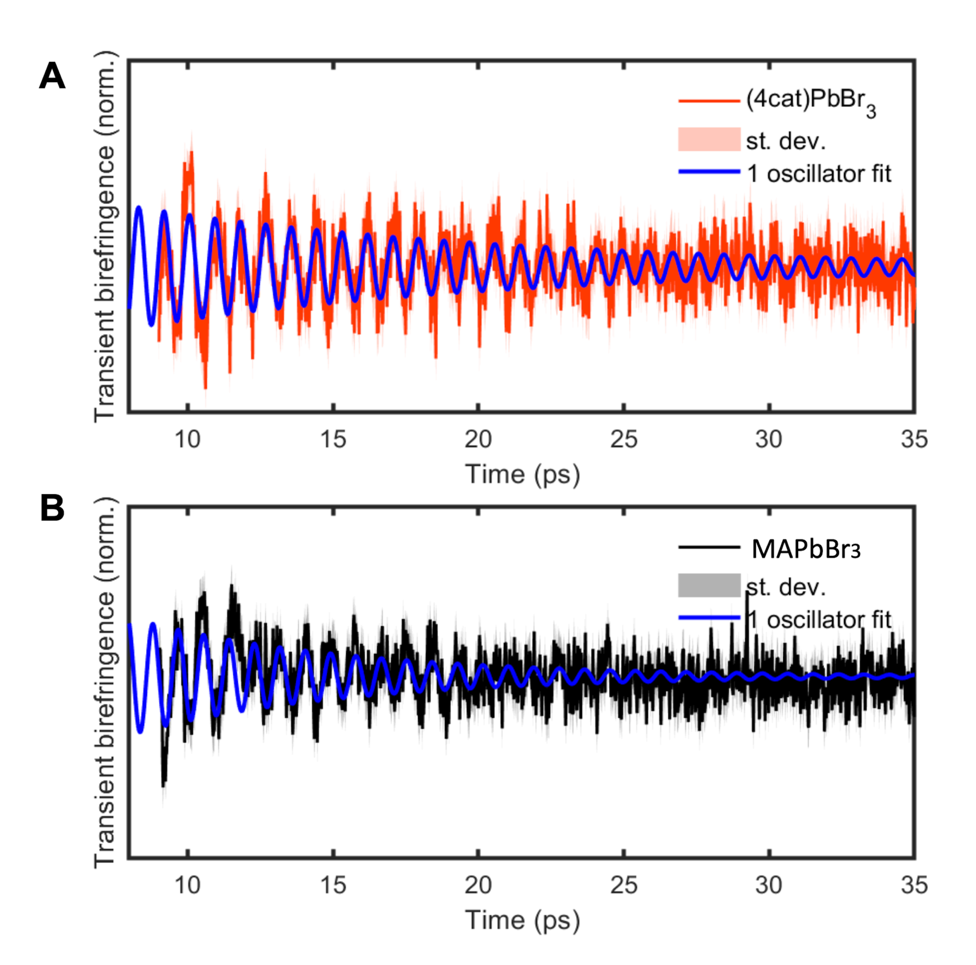
*

**Figure S8. Exemplary time-domain fits of the transient birefringence** for (A) (4cat)PbBr_3_ and (B) MAPbBr_3_ at 80 K.

**Section S8. Fluence measurements**


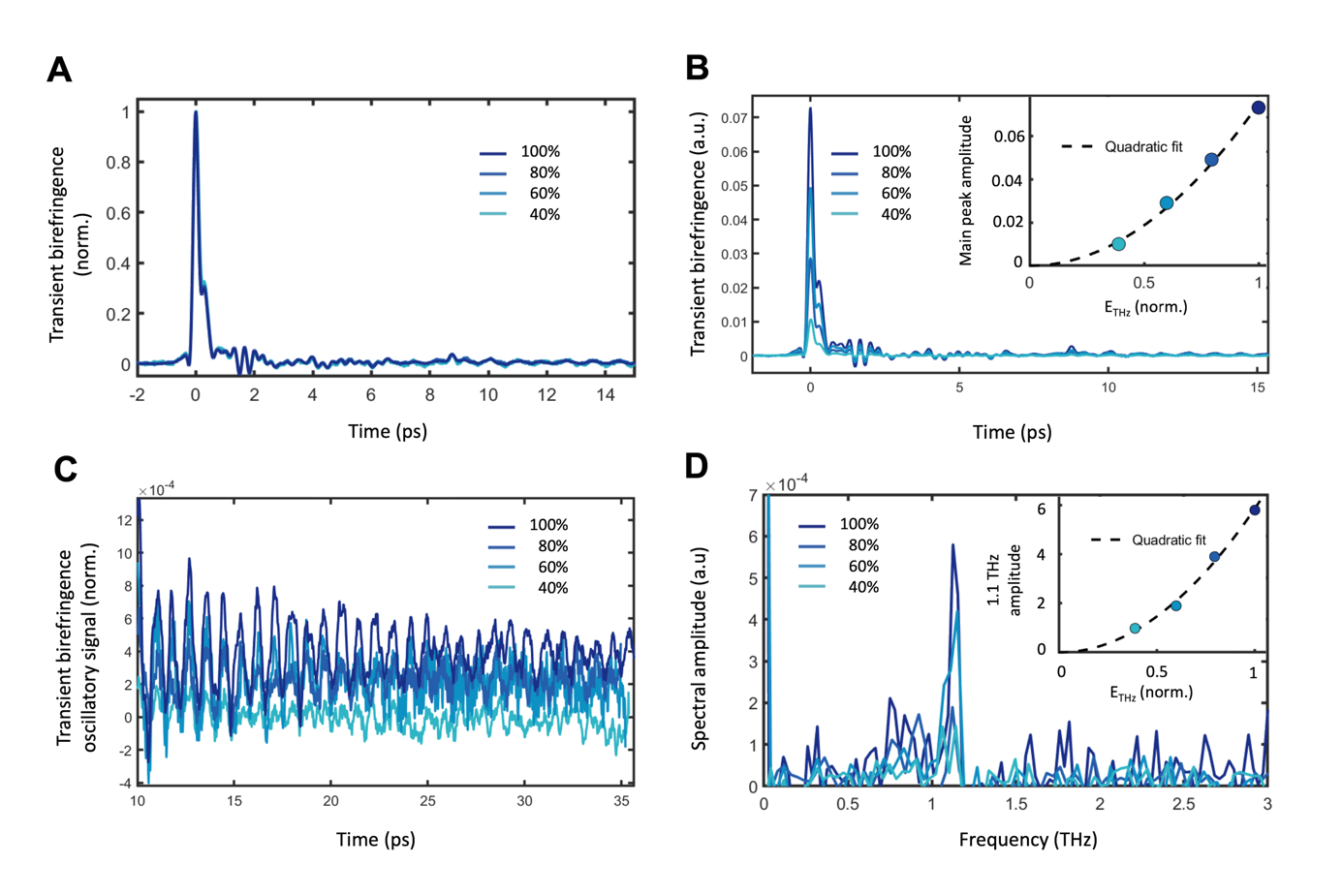


**Figure S9. THz pump fluence-dependent nonlinear excitation of Raman-active phonons in (4cat)PbBr_3_ thin films at 80 K:** Normalized (A) and unnormalized (B) TKE data acquired at 80 K under various THz pump fluences for (4cat)PbBr_3_ thin films of 350 nm thickness. The inset shows that the main peak scales quadratically with the square of the THz field. (C) Transient birefringence of the oscillatory signal normalized, for various fluences and (D) its Fourier Transform. The inset highlights that the dominating oscillatory amplitudes scale quadratically with the THz electric field.

**Section S9. Reproducibility of TKE signals**


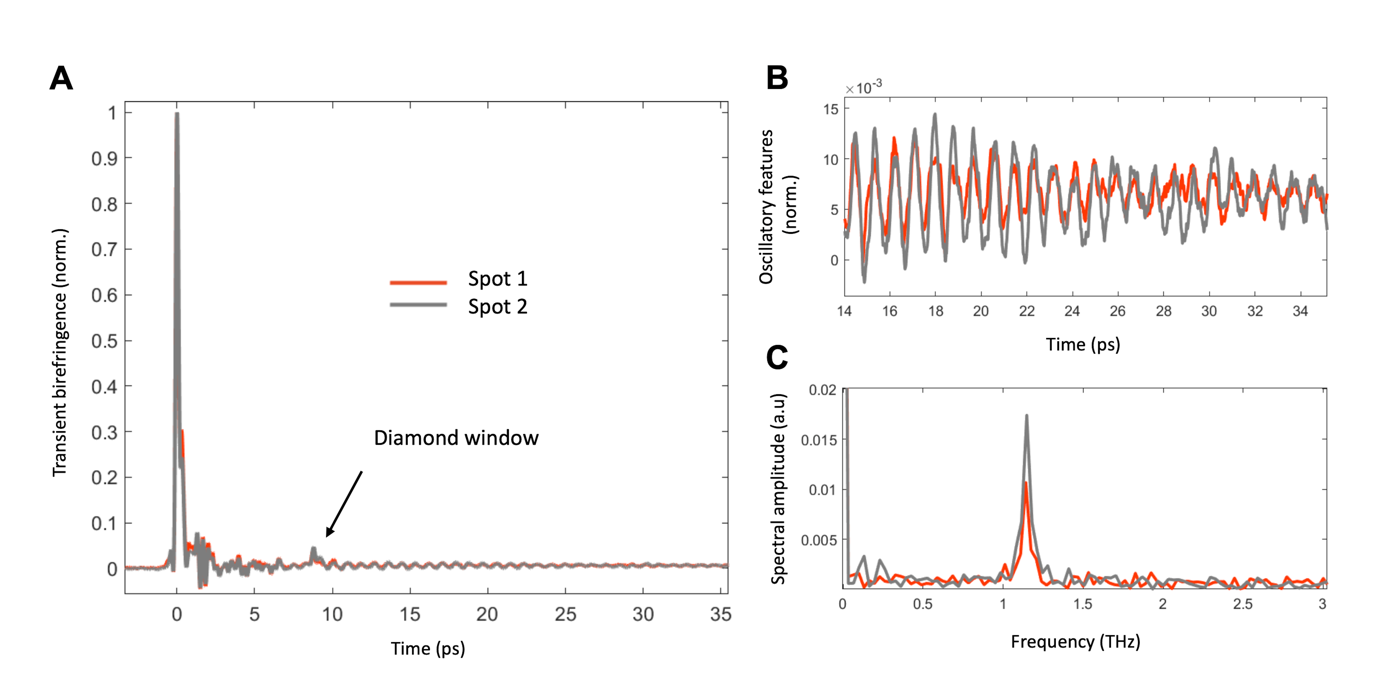


**Figure S10.** **Reproducibility of (4cat)PbBr_3_ signal:** (A) THz-induced transient birefringence of (4cat)PbBr_3_ thin film, 350 nm thick, at different days and on different spots. Measurements were taken at 4-month intervals, showing a certain stability of the thin film. (B) Oscillatory signal of the transient birefringence, from 14 to 45 ps. (C) Fourier transform of the oscillatory signals, where 1.1 THz phonon mode clearly appear and confirms its high reproducibility.

Notably, we observe no discernible signal from the BK7 glass substrate, except for the initial picosecond range (from 0 to 4ps as shown in Figure S7.B). The slight peak observed at approximately 9 ps in Figure S10 is attributed to the diamond windows used in the cryogenic setup. Between measurements, the samples were stored in vacuum desiccator.

**Section S10. Raman spectra of (4cat)PbBr_3_ single crystal**

The structural resolution of multi-cation perovskites is challenging due to the presence of multiple organic cations, making it difficult to fully solve the complete crystal structure in any publication. Therefore, to confirm the absence of phase transitions in the investigated temperature range, we performed complementary measurements by static Raman scattering of the (4cat)PbBr₃ single crystals presented in Figure S11.C. The spectra exhibit slight peak broadening with increasing temperature, likely due to increased anharmonic scattering of the corresponding phonon modes. Abrupt peak shifts or a change in the number of the observed peaks (i.e. splitting) would be indicative of a phase transition and corresponding distortions of the static crystal structure and changes of symmetry ^[26,27]^. However, in our case, we do not observe any significant spectral changes, only minor shifts and small broadening, of the Raman peaks, corroborating an absence of crystal phase transitions from 80 K to 300 K.

**
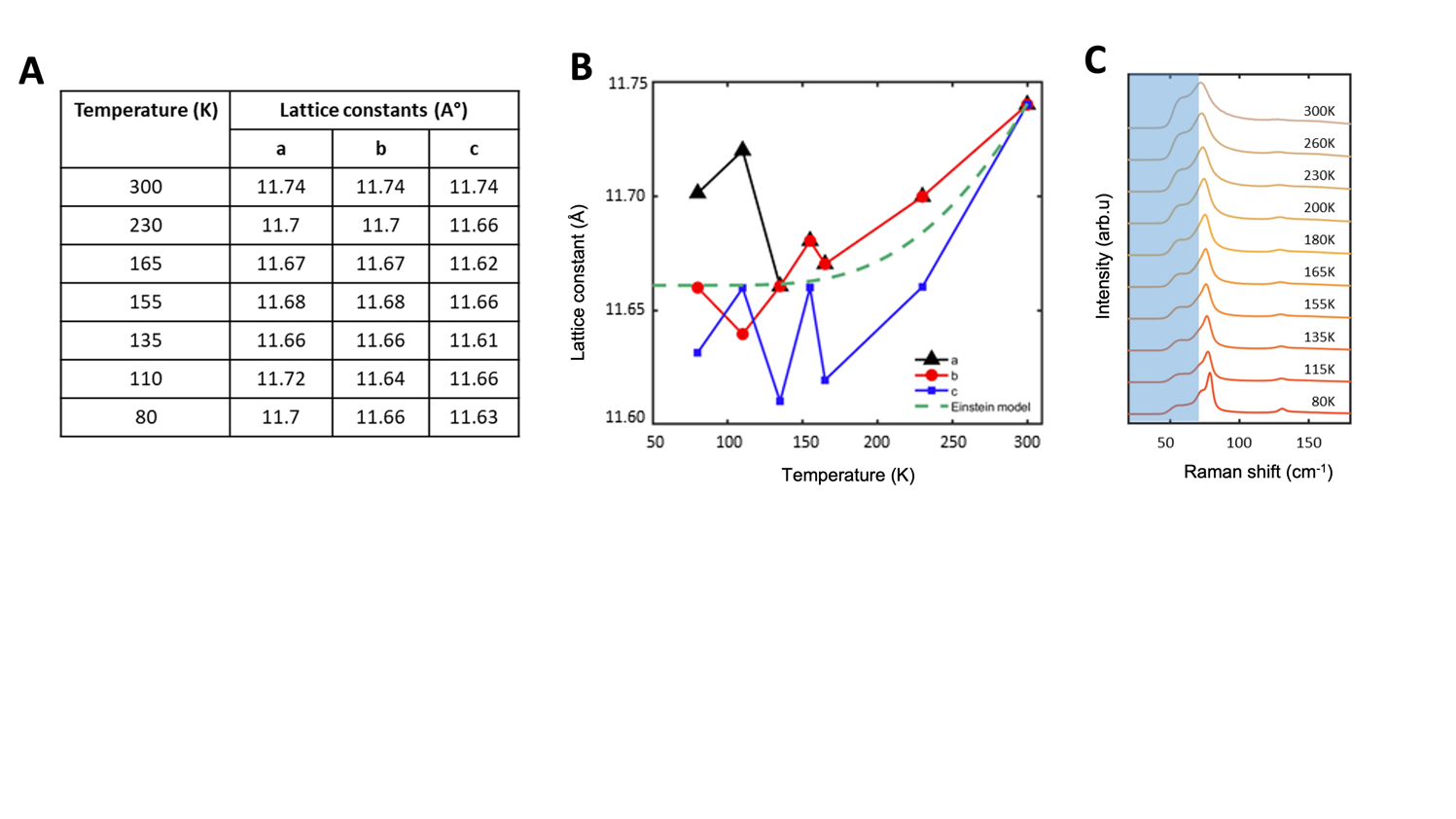
Figure S11. (4cat)PbBr_3_ single crystal characterization** **A)** Exact lattice constants determined from single crystal XRD measurements across various temperatures. B) Temperature-dependent XRD plot confirms the persistence of the cubic perovskite phase down to 80 K. C) Static Raman spectra shows slight and continuous blueshift (6.28 cm^-1^) from 72.38 cm^-1^ at 300 K to 78.66 cm^-1^ at 80 K of the cage modes excluding an abrupt phase transition in agreement with the single crystal X-Ray diffraction XRD results.

**Section S11. Einstein model of lattice thermal expansion**

The temperature dependence of the cubic lattice constant determined from the XRD measurements was fit with the following Einstein model:

$a\left( T \right)=a_{0}+a_{1}\Theta\left( \frac{1}{e^{\frac{\Theta}{T}}-1} \right)$*(S13)*

The fit was performed including all the data points for all *a,b*, and *c* lattice constants (assuming an average lattice constant in the *a,b,c* directions). The linear thermal expansion coefficient $\alpha$ is defined as

$\alpha\left( T \right)=\frac{1}{a_{RT}}\frac{da(T)}{dT}$*(S14)*

where $a_{RT}$ is the lattice constant at room temperature. We calculate $\alpha\left( T \right)$ using the derivative of the fit of Equation S14 to the XRD data as $\frac{da(T)}{dT}$ (Figure S12).


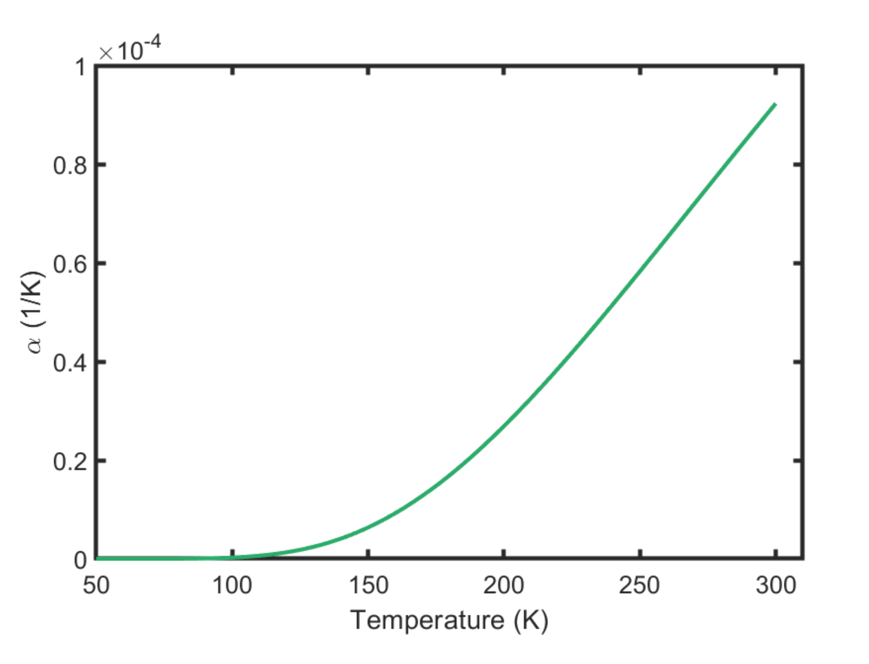


**Figure S12.** Thermal expansion coefficient of (4cat)PbBr_3_ calculated based on the Einstein model fit to the XRD-determined lattice constants.

**Section S12. Thermal expansion contribution to phonon frequency shift**

The thermal expansion contribution to the frequency shift (assuming cubic lattice $a_{a}=a_{b}=a_{c}=a$) can be expressed as:^[28]^

$\Delta_{0}\left( T \right)=-f_{0}\gamma\int_{0}^{T} 3\alpha\left( T^{'} \right)dT^{'}$*(S15)*

where $f_{0}$is the harmonic frequency of the mode without considering temperature effects and $\gamma$ is the Grüneisen parameter. We approximate the unknown value of this parameter for (4cat)PbBr_3_ with the literature value for FAPbBr_3_ $\gamma=1.8.$^[29]^

**Section S13. XRD on thin films**


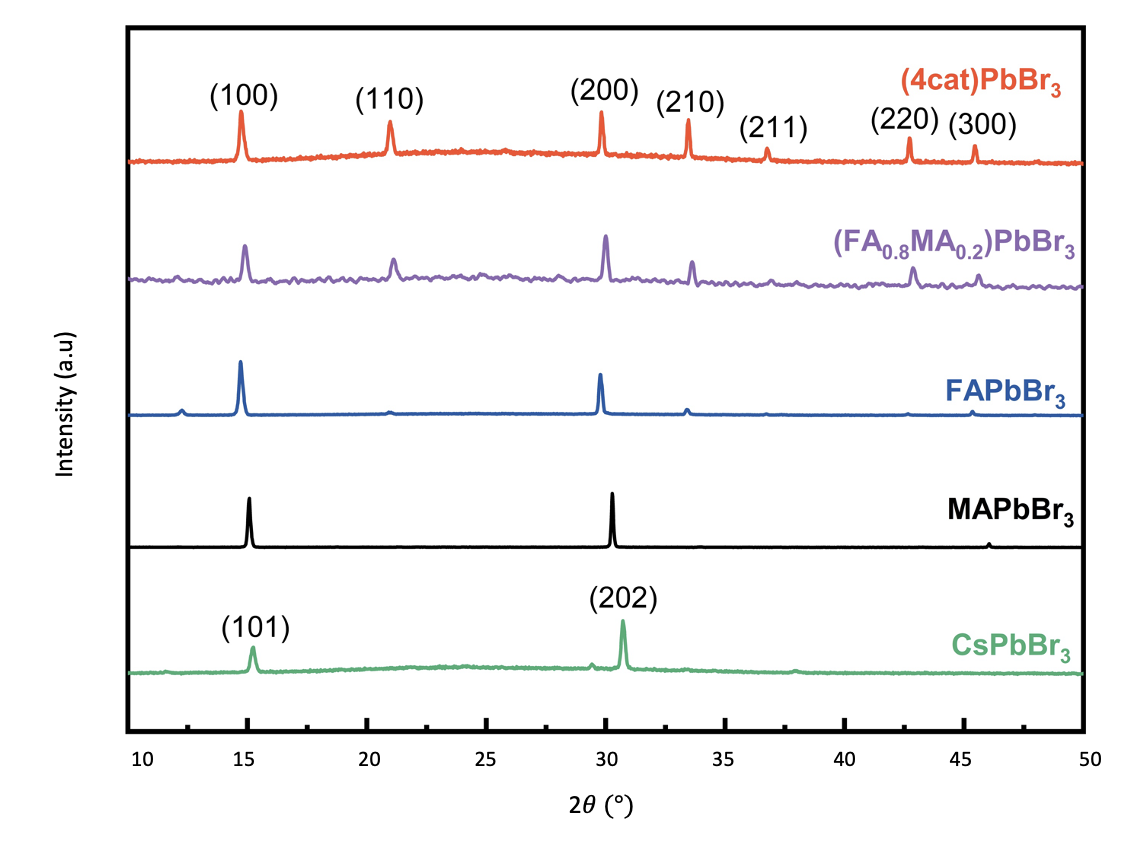


**Figure S13.** XRD results for perovskite thin films at room temperature that shows all films has cubic crystal structure according to the cubic plane peaks.

**Section S14. X-ray photoelectron spectroscopy (XPS):**

To establish a baseline for comparison, XPS spectra for FAPbBr₃, MAPbBr₃, and GAPbBr₃ were measured as a reference (Figure S14.A). The distinct features of MA^+^, FA^+^, and GA^+^ are clearly identified in the C 1s and N 1s core-level spectra, allowing us to observe the chemical changes induced in the perovskite structure when different A-site cations are introduced. To further identify the presence of Cs⁺ and GA⁺, a triple-cation perovskite without GA^+^, composed of (Cs_0.0515_MA_0.1615_FA_0.787_)PbBr_3_ were evaluated. The resulting XPS spectrum (Figure S14.B) revealed separable peaks corresponding to C MA 1s and C FA 1s, along with the appearance of the Cs 3d_5/2_ peak. In contrast, the (4cat)PbBr₃ XPS spectrum in Figure S14.C displays emission lines for all expected organic cations—MA, FA, and GA—at 286.4 eV, 288.4 eV, and 289.1 eV, respectively. This confirms that upon the addition of GA, a shift in the chemical bonding is observed, which in agreement with literature ^[21]^ is considered a clear proof of a changed local environment of C 1s. Given that the signals originate from a similar probing depth, the peak intensities can be confidently correlated with the overall composition. Consequently, the increased intensity of the C-H 1s peak in the (4cat)PbBr₃ film further corroborates the presence of GA, as it aligns with the higher C-H 1s peak observed in the GAPbBr₃ film. Furthermore, we observed a narrowing of the C FA 1s peak in the (4cat)PbBr₃ sample (≈ 90 meV), suggesting subtle changes in the chemical environment around the FA cation, which we interpret as indicative of improved homogeneity or bonding characteristics in the quadruple-cation perovskite^[22]^. In contrast identification of the weak GA component alongside the FA component in the N 1s spectrum is more challenging than in the C 1s spectrum due to the generally broader peaks. In fact, given the significant overlap of the FA and GA peaks in the N 1s region, we observed a FWHM of 1.08 eV for the triple cation and 1.11 eV for the quadruple cation, which is consistent with the addition of 1.5% GA.

It is important to note that the stoichiometry of the precursor solution differs from the final thin film composition. Consequently, the stoichiometries reported in literature, often deviate from a 1:1:3 ratio for cation:lead:halide^[23]^. By integrating the peak areas in the XPS spectra, determined the relative atomic percentages of the elements. Note, that this is only meaningful for a probing depth of at most 10 nm of the perovskite film, due to the limiting escape depth of the photoelectrons.


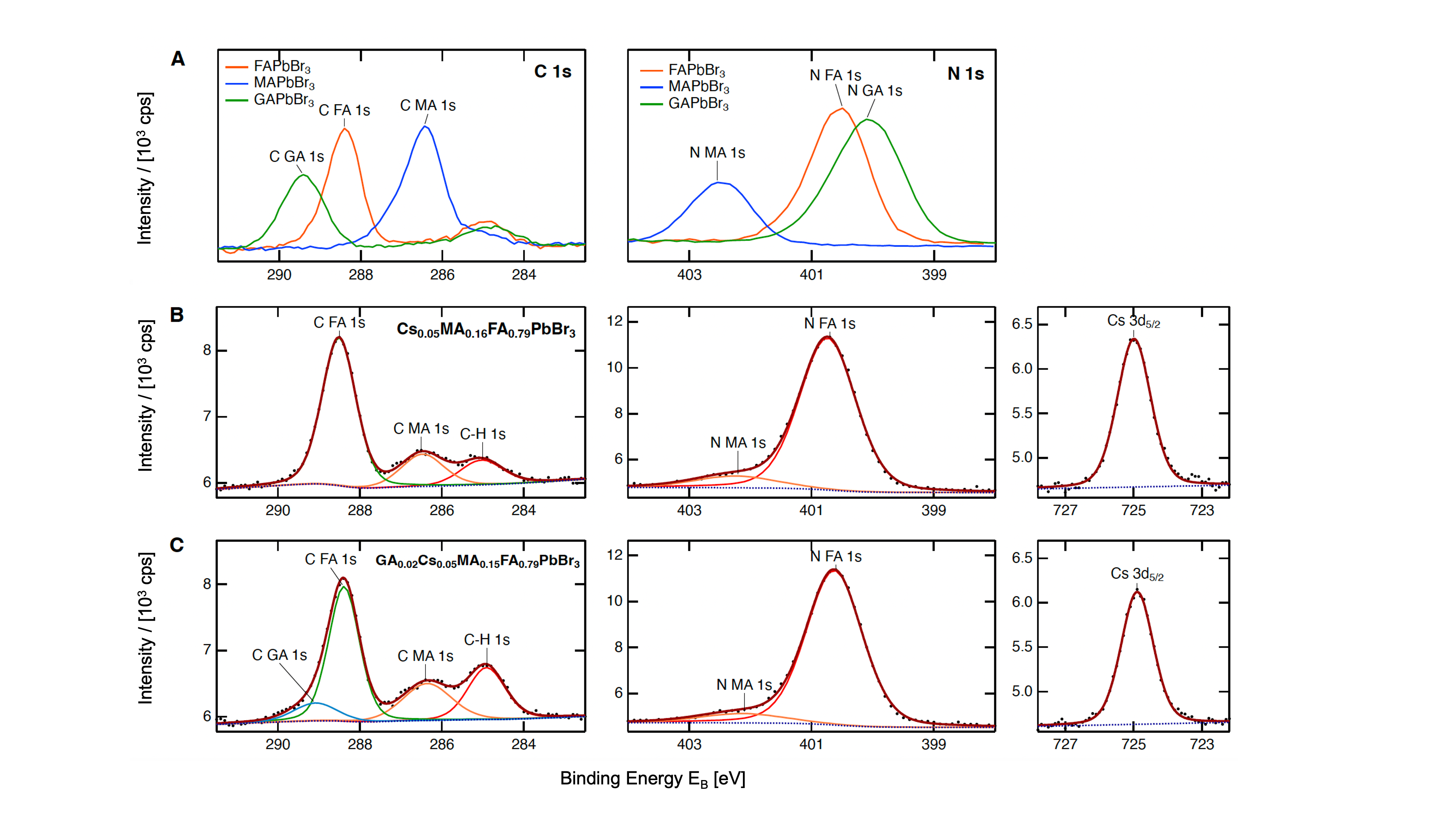


**Figure S14**. High resolution X-ray photoelectron spectroscopy (XPS) A) C 1S and N 1S core-level spectra for MAPbBr_3_, FAPbBr_3_, and GAPbBr_3_ thin films. C 1S, N 1S, and Cs 3d_5/2_ of B) (Cs_0.0515_MA_0.1615_FA_0.787_)PbBr_3_ thin film and C) (GA_0.015_Cs_0.046_MA_0.152_FA_0.787_)PbBr_3_ thin film**.**

**Section S15. Exploring multiple phonon modes in (4cat)PbBr_3_: implications for A-site cation diversity**

**
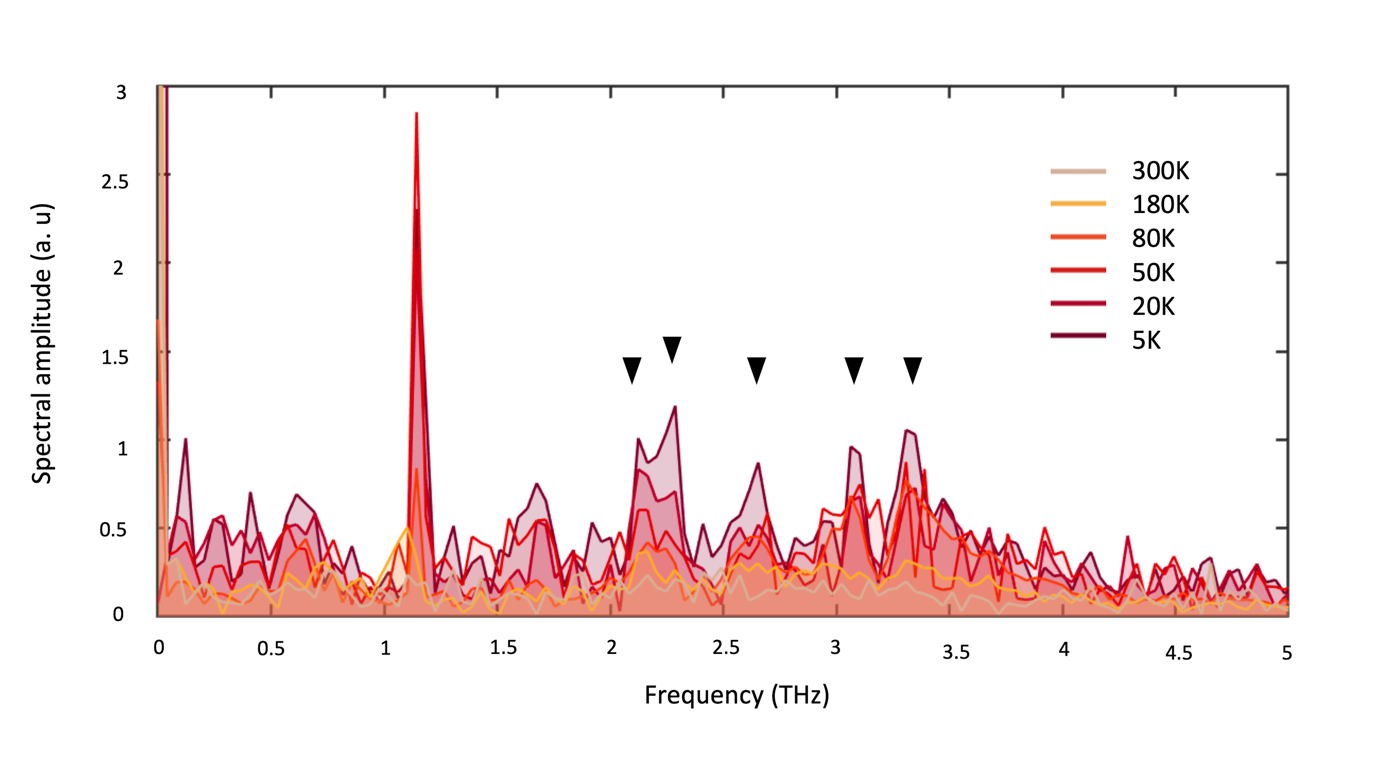
Figure S15.** Temperature-dependent FT of the (4cat)PbBr_3_ thin film TKE oscillatory signal presented in Figure 3C, for cut-off time 1.1 ps > *t* > 35 ps. In addition to the sharp phonon mode at 1.1 THz, 5 shorter-lived higher frequency peaks are observed (black arrows). These modes become clearer with decreasing temperature.

While the long-lived dynamic lattice response is dominated by the 1.1 THz mode (Figure 3C), at low temperatures we additionally witness short-lived modes in a Fourier transform of the signal cutoff after 1.5 ps (see Figure S15). These higher frequency modes at 70 cm^-1^ (2.1 THz), 73 cm^-1^ (2.2 THz), 86 cm^-1^ (2.6 THz), 103 cm^-1^ (3.1 THz), and 110 cm^-1^ (3.3 THz), become increasingly prominent as the temperature decreases, and their lifetimes increase as indicated by the decreasing FT peak width. It is worth noting that these short-lived modes are absent in the TKE response of MAPbBr_3_, FAPbBr_3_, and CsPbBr_3_ (SI Section S18) and therefore a clear fingerprint of the specific impact of the quadruple cation composition on the lattice dynamics. Investigations of the origin of other modes are left to further TKE and Raman scattering studies. To rule out effects from dispersive light propagation, thickness dependence measurements were also performed (SI Section S19).

The complex dynamics influencing the behavior of the 1.1 THz octahedral twist mode below 80 K may be related to the observed emergence of multiple higher-frequency vibrational modes at those lowest temperatures. (4cat)PbBr_3_ and MAPbBr_3_ show the same long-lived phonon vibration at 1.1 THz, nevertheless only (4cat)PbBr_3_ additionally exhibits few short-lived vibration modes between 2.2 THz and 3.3 THz (see Figure S17). This is the first observation of these low-temperature modes, as temperature-dependent low-frequency Raman spectra below 60 cm⁻¹ of (4cat)PbBr_3_ could not be obtained, yet. Furthermore, these findings raise intriguing questions about the relationship between the coexistence of these relatively short-lived modes, the long lifetime of the 1.1 THz mode in (4cat)PbBr_3_, and the similarity of the 1.1 THz mode to MAPbBr_3_ at temperatures below 80 K. The complexity of the A-site cation composition in (4cat)PbBr_3_ may underpin the presence of these short-lived phonon modes, although further investigation is required to determine their precise nature. Hypothetically, the short-lived phonon modes may represent hybrid modes, which emerge as a consequence of the coupling between inorganic cage vibrations and cation motion, e.g. similar to MA lurching between 2.1 THz to 3 THz in MAPbBr_3_.^[26]^ Moreover, it is conceivable that the lifetime of these higher frequency modes is also increased in (4cat)PbBr_3_, thereby offering the opportunity to observe them.

**Section S16. Temperature dependence of phonon linewidth *Γ*.**

The temperature dependence of *Γ* is fitted by a simple model assuming only the decay of the initial optical phonon into two (cubic anharmonicity, Equation S16) or three (quartic anharmonicity, Equation S17) equally energetic phonons:

$\Gamma_{3 ph}\left( T \right)=\Gamma_{0}+A \left( 1+\frac{2}{e^{\frac{hf}{2k_{B}T}}-1} \right)$*(S16)*

$\Gamma_{4 ph}\left( T \right)=\Gamma_{0}+B \left( 1+\frac{3}{e^{\frac{hf}{3k_{B}T}}-1}+\frac{3}{\left( e^{\frac{hf}{3k_{B}T}}-1 \right)^{2}} \right)$*(S17)*

where $f$ is the initial optical phonon frequency (we can assume it as a parameter in the $\Gamma\left( T \right)$ fitting as the change of $f$ with temperature is relatively small^[32]^, $\Gamma_{0}$ the temperature-independent broadening due to impurity scattering, the anharmonic constants $A,B$ correspond to the intrinsic linewidths at$T=0,$ and$k_{B}$is the Boltzmann constant.

The temperature-dependent phonon linewidths calculated from the damping times extracted from the time-domain fits were fit with the models described by equation (S16) (describing only 3 phonon processes) and (S17) (describing only 4 phonon processes). The obtained fit parameters are listed in table S3. The mode frequency was fixed at values obtained from the frequency shift fit (see above) to reduce the number of fit parameters.

**Table S3.** Fit parameters extracted from the anharmonic decay models given by equation (S16) and (S17).

| Model | $\Gamma_{0}$ [THz] | $A$ [THz] | $B$ [THz] | $f$ [THz] |
| --- | --- | --- | --- | --- |
| 3-phonon scattering | 0.0104 | 0.0025 | - | 1.1589 (fixed) |
| 4-phonon scattering | 0.0140 | - | 1.72∙10^-4^ | 1.1532 (fixed) |

**Section S17. 3- and 4-phonon anharmonic scattering model of phonon frequency shift and lifetime as a function of temperature for the 1.1 THz mode**

The shift of phonon frequency due to cubic anharmonicity for the symmetric Klemens phonon-phonon scattering channel is expressed as:

$\Delta_{anh,3}\left( T \right)=C \left( 1+\frac{2}{e^{\frac{hf}{2k_{B}T}}-1} \right)$*(S18)*

and for the symmetric decay due to quartic anharmonicity:^[30]^

$\Delta_{anh,4}\left( T \right)=D \left( 1+\frac{3}{e^{\frac{hf}{3k_{B}T}}-1}+\frac{3}{\left( e^{\frac{hf}{3k_{B}T}}-1 \right)^{2}} \right)$*(S19)*

where *C, D* are anharmonic constants, $k_{B}$ is the Boltzmann constant, $h$ is the Planck constant and $f$ the phonon frequency.

To model the temperature-dependent frequency shifts, a constant offset plus the anharmonic term ${(f}_{0}+\Delta_{anh}\left( T \right))$ (where $\Delta_{anh}\left( T \right)= \Delta_{anh,3}\left( T \right)$ for 3-phonon scattering or $\Delta_{anh}\left( T \right)= \Delta_{anh,4}\left( T \right)$ for 4-phonon scattering) was fit to the experimentally observed frequencies after subtracting the lattice expansion term ($f\left( T \right)-\Delta_{0}\left( T \right))$. The obtained fit parameters are listed in table S4.

**Table S4.** Fit parameters extracted from the anharmonic decay models given by (S18) and (S19).

| **Model** | $\boldsymbol{f}_{\boldsymbol{0}}$ **[THz]** | $\boldsymbol{C}$ **[THz]** | $\boldsymbol{D}$ **[THz]** |
| --- | --- | --- | --- |
| 3-phonon scattering | 1.1589 | -0.0027 | - |
| 4-phonon scattering | 1.1532 | - | -1.57∙10^-4^ |

Figure 4B compares the observed phonon frequency $f\left( T \right)$, the estimated frequency shift contribution from the thermal expansion and the total shift including $\Delta_{0}\left( T \right)$ and the phonon-phonon scattering terms. Clearly, lattice expansion alone cannot explain the experimental trend.

While a full analysis should include both three and four phonon scattering and account for example for possible asymmetric down-conversion processes^[28]^ (the generalized Ridley channel^[31]^), with our simple ballpark model we only want to qualitatively demonstrate the predicted influence of the different anharmonic processes.

***
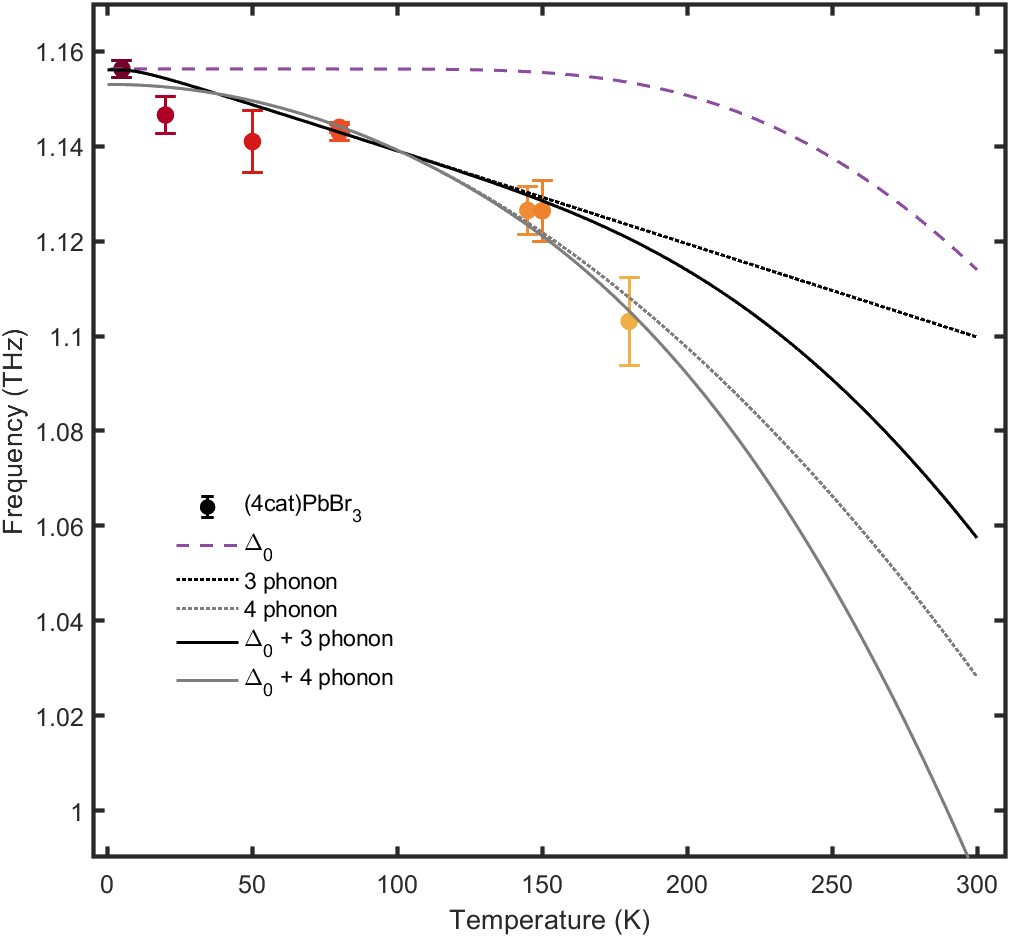
***

**Figure S16. Lattice expansion and three-phonon and four-phonon contributions to the frequency shift of the 1.1 THz mode in (4cat)PbBr_3_:** The points correspond to frequencies based on the time-domain fits to the experimental data. The dashed line indicates the lattice expansion term Δ_0_. The black and grey dotted lines and three-phonon and four-phonon contributions calculated for the symmetric scattering channel considering only three phonon, or only 4 phonon processes, respectively. Solid lines indicate the fits including both lattice expansion and phonon-phonon scattering contributions for three-phonon and four-phonon scattering models.

**Section S18. Short lived modes all thin films 80 K**

**
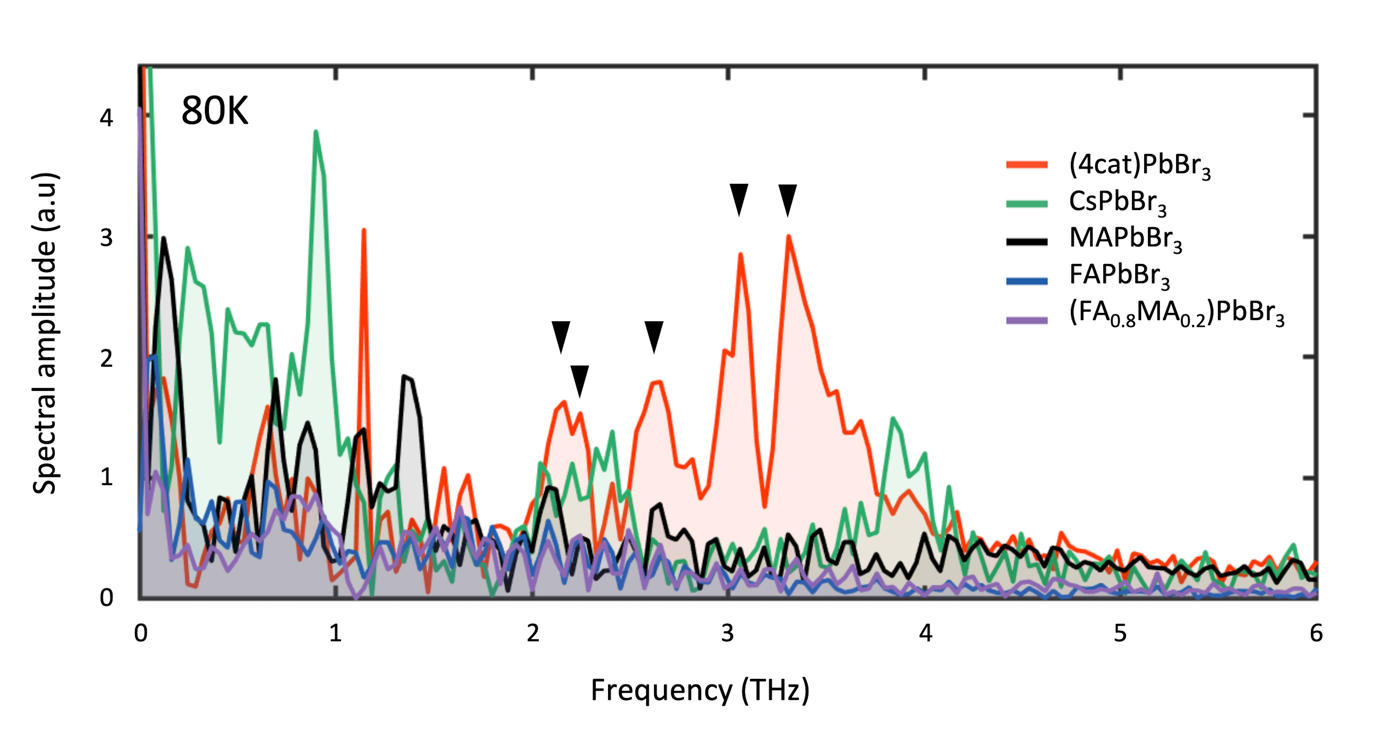
**

**Figure S17.** Transform of the (4cat)PbBr_3_, CsPbBr_3_, MAPbBr_3_, FAPbBr_3_ and (FA_0.8_MA_0.2_)PbBr_3_ thin films TKE oscillatory signal at 80K of Figure 3A, for 1.1 ps > t > 35 ps. In addition to the sharp phonon mode at 1.1 THz, the five other shorter-lived frequency peaks of (4cat)PbBr_3_ are marked with black arrows. They do not appear in other A-site cation compositions.

**Section S19. Thickness dependence of (4cat)PbBr3 thin films**


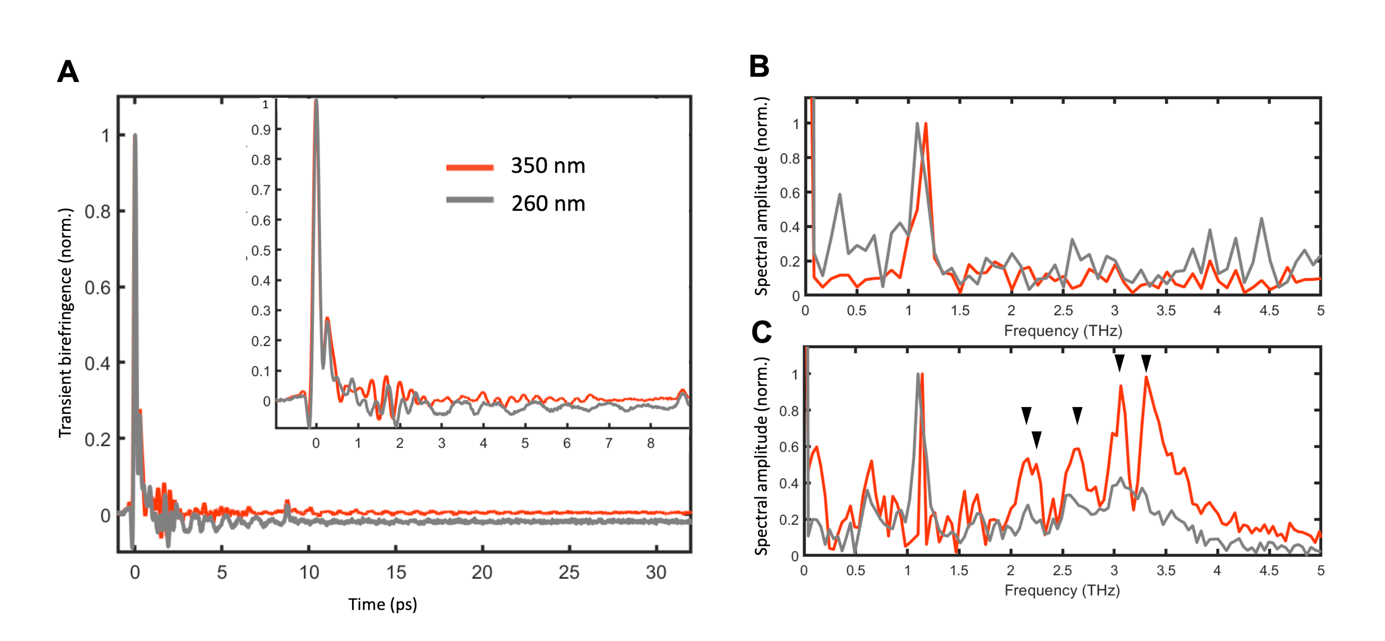


**Figure S18.** **Thickness dependence of (4cat)PbBr_3_ thin films:** Thickness dependence of the (4cat)PbBr3 thin film, with film thicknesses denoted as 350 nm (purple) and 260 nm (blue): (A) Transient birefringence and Fourier transform after two different cutoff in the *t* direction at 1.1 ps (B) and at (C) 12.68 ps. The different cutoffs in the *t*-direction provide insights into the material's temporal characteristics. Remarkably, both thin films exhibit 5 short-lived oscillations with closely matched frequencies (black triangles), unequivocally demonstrating that these oscillations are not artifacts but rather inherent properties of the (4cat)PbBr_3_ material.

To rule out effects from dispersive light propagation or Fabry-Pérot fringes, which may both cause oscillatory Kerr effect features on picosecond timescales,^[33,34]^ we conduct TKE experiment two different (4cat)PbBr_3_ thin film thicknesses: 350 nm and 260 nm (both on 500 μm-thick BK7 glass substrate). Despite differences in the overall TKE shape (see Figure S18.A), the TKE spectra in Figure S18.B confirm that long-lived oscillatory TKE features are independent of the samples thickness within our experimental frequency resolution. Also, the spectra of the short-lived oscillations indicate similar modulation frequencies (see Figure S18.C). This provides further evidence that also the short-lived coherence is related to additional phonon modes, likely strongly influenced by the A-site cation chemical composition.

**Supporting information References**

[1] M. I. Saidaminov, A. L. Abdelhady, B. Murali, E. Alarousu, V. M. Burlakov, W. Peng, I. Dursun, L. Wang, Y. He, G. Maculan, A. Goriely, T. Wu, O. F. Mohammed, O. M. Bakr, *Nat Commun* **2015**, *6*, 7586.

[2] Z. Yang, B. H. Babu, S. Wu, T. Liu, S. Fang, Z. Xiong, L. Han, W. Chen, *Review on Practical Interface Engineering of Perovskite Solar Cells: From Efficiency to Stability*, Vol. 4, Wiley-VCH Verlag, **2020**.

[3] F. Ünlü, E. Jung, S. Öz, H. Choi, T. Fischer, S. Mathur, In *Perovskite Solar Cells*, Wiley, **2021**, pp. 1–31.

[4] A. S. Subbiah, S. Agarwal, N. Mahuli, P. Nair, M. van Hest, S. K. Sarkar, *Adv Mater Interfaces* **2017**, *4*.

[5] B. Slimi, M. Mollar, I. Ben Assaker, A. Kriaa, R. Chtourou, B. Marí, *Monatshefte für Chemie - Chemical Monthly* **2017**, *148*, 835.

[6] P Wurfel, *Journal of Physics C: Solid State Physics* **1982**, *15*, 3967.

[7] Z. Liu, L. Krückemeier, B. Krogmeier, B. Klingebiel, J. A. Márquez, S. Levcenko, S. Öz, S. Mathur, U. Rau, T. Unold, T. Kirchartz, *ACS Energy Lett* **2019**, *4*, 110.

[8] W. Peng, C. Aranda, O. M. Bakr, G. Garcia-Belmonte, J. Bisquert, A. Guerrero, *ACS Energy Lett* **2018**, *3*, 1477.

[9] A. Bou, A. Pockett, D. Raptis, T. Watson, M. J. Carnie, J. Bisquert, *J Phys Chem Lett* **2020**, *11*, 8654.

[10] S. M. Abdulrahim, Z. Ahmad, J. Bahadra, N. J. Al-Thani, *Nanomaterials* **2020**, *10*, 1635.

[11] M. T. Khan, M. Salado, A. Almohammedi, S. Kazim, S. Ahmad, *Adv Mater Interfaces* **2019**, *6*.

[12] T. Bandara, B. E. Mellander, *Ionic liquids: theory, properties, new approaches* **2011**, *17*, 383.

[13] T. Q. Nguyen, C. Breitkopf, *J Electrochem Soc* **2018**, *165*, E826.

[14] G. Garcia-Belmonte, A. Munar, E. M. Barea, J. Bisquert, I. Ugarte, R. Pacios, *Org Electron* **2008**, *9*, 847.

[15] M. Frenzel, M. Cherasse, J. M. Urban, F. Wang, B. Xiang, L. Nest, L. Huber, L. Perfetti, M. Wolf, T. Kampfrath, X.-Y. Zhu, S. F. Maehrlein, *Sci Adv* **2023**, *9*.

[16] M. Mączka, J. A. Zienkiewicz, M. Ptak, *The Journal of Physical Chemistry C* **2022**, *126*, 4048.

[17] F. Wang, Y. Fu, M. E. Ziffer, Y. Dai, S. F. Maehrlein, X.-Y. Zhu, *J Am Chem Soc* **2021**, *143*, 5.

[18] A. C. Ferreira, S. Paofai, A. Létoublon, J. Ollivier, S. Raymond, B. Hehlen, B. Rufflé, S. Cordier, C. Katan, J. Even, P. Bourges, *Commun Phys* **2020**, *3*, 48.

[19] D. J. Kubicki, D. Prochowicz, A. Hofstetter, P. Péchy, S. M. Zakeeruddin, M. Grätzel, L. Emsley, *J Am Chem Soc* **2017**, *139*, 10055.

[20] J. Gong, M. Yang, X. Ma, R. D. Schaller, G. Liu, L. Kong, Y. Yang, M. C. Beard, M. Lesslie, Y. Dai, B. Huang, K. Zhu, T. Xu, *J Phys Chem Lett* **2016**, *7*, 2879.

[21] S. Gharibzadeh, B. Abdollahi Nejand, M. Jakoby, T. Abzieher, D. Hauschild, S. Moghadamzadeh, J. A. Schwenzer, P. Brenner, R. Schmager, A. A. Haghighirad, L. Weinhardt, U. Lemmer, B. S. Richards, I. A. Howard, U. W. Paetzold, *Adv Energy Mater* **2019**, *9*.

[22] A. Kulkarni, R. Sarkar, S. Akel, M. Häser, B. Klingebiel, M. Wuttig, S. Wiegand, S. Chakraborty, M. Saliba, T. Kirchartz, *Adv Funct Mater* **2023**, *33*.

[23] H.-K. Si, A. F. Musa, T.-S. Su, T.-C. Wei, *J Mater Chem C Mater* **2024**, *12*, 6341.

[24] X. Zhang, W. Zhou, X. Chen, Y. Chen, X. Li, M. Wang, Y. Zhou, H. Yan, Z. Zheng, Y. Zhang, *Adv Energy Mater* **2022**, *12*.

[25] N. De Marco, H. Zhou, Q. Chen, P. Sun, Z. Liu, L. Meng, E.-P. Yao, Y. Liu, A. Schiffer, Y. Yang, *Nano Lett* **2016**, *16*, 1009.

[26] A. M. A. Leguy, A. R. Goñi, J. M. Frost, J. Skelton, F. Brivio, X. Rodríguez-Martínez, O. J. Weber, A. Pallipurath, M. I. Alonso, M. Campoy-Quiles, M. T. Weller, J. Nelson, A. Walsh, P. R. F. Barnes, *Physical Chemistry Chemical Physics* **2016**, *18*, 27051.

[27] M. Mączka, M. Ptak, *Solids* **2022**, *3*, 111.

[28] R. Cuscó, E. Alarcón-Lladó, J. Ibáñez, L. Artús, J. Jiménez, B. Wang, M. J. Callahan, *Phys Rev B* **2007**, *75*, 165202.

[29] R. Mayengbam, J. T. Mazumder, *Int J Energy Res* **2022**, *46*, 17556.

[30] M. Balkanski, R. F. Wallis, E. Haro, *Phys Rev B* **1983**, *28*, 1928.

[31] B. K. Ridley, *Journal of Physics: Condensed Matter* **1996**, *8*, L511.

[32] T. Kohmoto, M. Masui, M. Abe, T. Moriyasu, K. Tanaka, *Phys Rev B* **2011**, *83*, 064304.

[33] S. F. Maehrlein, P. P. Joshi, L. Huber, F. Wang, M. Cherasse, Y. Liu, D. M. Juraschek, E. Mosconi, D. Meggiolaro, F. De Angelis, X.-Y. Zhu, *Proceedings of the National Academy of Sciences* **2021**, *118*.

[34] L. Huber, S. F. Maehrlein, F. Wang, Y. Liu, X.-Y. Zhu, *J Chem Phys* **2021**, *154*.
